# Blood Relatives: Linking Evolutionary History and Conservation of Medicinal Leeches (*Hirudo* spp.)

**DOI:** 10.1111/eva.70273

**Published:** 2026-05-28

**Authors:** Gianluca Lombardo, Alessandro Alvaro, Andrea De Benedictis, Marta Cavallini, Milo Manica, Edward C. Netherlands, Laura Pulze, Nicolò Baranzini, Annalisa Grimaldi, Francesco Acquati, Giorgio Binelli

**Affiliations:** ^1^ Department of Biotechnology and Life Sciences (DBSV) University of Insubria Varese Italy; ^2^ Department of Biosciences University of Milan Milan Italy; ^3^ Gruppo Insubrico di Ornitologia l Clivio Italy; ^4^ Tutela anfibi basso Verbano Sesto Calende Italy; ^5^ Department of Zoology and Entomology Bloemfontein South Africa

**Keywords:** conservation genetics, cryptic species, glacial refugia, *Hirudo*, medicinal leech, phylogeography, Pleistocene refugia, taxonomy

## Abstract

Medicinal leeches of the genus *Hirudo* are both historically important in medicine and increasingly studied as models in ecology and biomedicine. However, the taxonomy, population structure and evolutionary history of this genus remain partially unresolved. Here, we present the most comprehensive phylogenetic analysis of the *Hirudo* complex to date, incorporating 350 *COX1* sequences (103 newly generated) and 247 complete mitogenomes from across the distribution range. In *Hirudo verbana*, three major clades corresponding to Iberian (A), Western (B) and Eastern (C) haplogroups were found, with sub‐haplogroups reflecting glacial refugia and Pleistocene expansions. Diversity was highest in Eastern populations, consistent with Anatolia and the Black Sea basin acting as long‐term refugia. Bayesian divergence dating indicates that the genus originated in the mid‐Miocene (~15 Mya), with an early split of *H. nipponia* in East Asia. Subsequent radiations shaped the Western Eurasian clade, with *H. sulukii* diverging ~9.9 Mya, *H. orientalis* splitting from European lineages ~6.4 Mya and differentiating ~440 kya, and recent radiations of *H. verbana*, *H. medicinalis* and *H. troctina* occurring during the late Pleistocene. Notably, nucleotide diversity within *H. nipponia* (~8.8%) and deep divergence among its haplogroups suggest the presence of multiple cryptic species, with *H. tianjinensis* clustering within these lineages. We also molecularly identified several Italian specimens as *Haemopis sanguisuga* or a potential new *Dina* species. These findings emphasise the importance of taxonomic clarity and genetic monitoring to guide conservation and prevent overexploitation of *Hirudo* leeches, ensuring the long‐term sustainability and biomedical utility of these ecologically significant annelids.

## 1 Introduction

Medicinal leeches of the genus *Hirudo* Linnaeus, 1758 have recently had a comeback in medicine and research context, aside from their bloodletting practices (Montinari and Minelli 2022), becoming model organisms for biomedicine (Baranzini et al. 2020; Lemke and Vilcinskas 2020), neurobiology (Boidin‐Wichlacz et al. 2012; Wagenaar 2015) and ecological studies (Weiskopf et al. 2018; Baranzini et al. 2022). In particular, their renewed clinical importance lies in the efficacy of their saliva, which contains over 200 bioactive compounds, including potent anticoagulants and anti‐inflammatory agents, used to treat localised venous congestion, osteoarthritis and promote tissue healing in reconstructive surgery (Hildebrandt and Lemke 2011). In addition, medicinal leeches are applied as non‐invasive tools in zoology and ecophysiology for blood sampling and pathogen surveillance, providing innovative approaches to wildlife conservation and monitoring animal health without causing stress to the target organisms (Wang et al. 2025).

The evolutionary history of the genus has also undergone major revisions in the last couple of decades, also thanks to the advent of next generation sequencing techniques, which allowed for the sequencing of the first complete genome and the construction of the first salivary transcriptome (Babenko et al. 2020). The *Hirudo* species complex has been historically treated as a single widely distributed European group named ‘medicinal leech’ (Kutschera and Elliott 2014) historically regarded as roughly 20 species (Moquin‐Tandon 1846), but this conception has been challenged in the past decades by molecular systematics analyses, which have clarified that the term ‘medicinal leech’ does not represent a single species, but indeed a Western Eurasian clade comprising different species that differ in morphology, ecology and range (Siddall et al. 2007; Utevsky et al. 2010; Trontelj and Utevsky 2012). Moreover, historical studies also point towards the linguistic confusion of the term ‘*Sanguisuga*’ (leech) and its derivatives in different European languages that could have played an important role in the classification confusion (Moquin‐Tandon 1846). Within this complex, the foremost species are: *H. medicinalis* (western/central Europe), *H. verbana* (Iberia, Turkey, central Europe and Caucasus), *Hirudo orientalis* (Transcaucasia–Iran–Central Asia), *H. troctina* (Maghreb, Sardinia and parts of Iberia) and the recently described, *H. sulukii*, from Turkey (Ceylan et al. 2021). An additional species, *Hirudo nipponia*, is present in East Asia and is discontinuous from the former species in distribution, with another species described in a small area of China named *H. tianjinensis* (Wang et al. 2022). All these species share a dorsoventrally flattened, segmented body with two suckers but have been distinguished at the morphological level by coloration and other traits. *H. medicinalis* is large (up to 20 cm), greenish‐brown with thin red dorsal stripes and has three jaws with approximately 100 teeth. *H. verbana* is more robust, featuring broad orange paramedian dorsal stripes and yellow‐green ventral coloration with black ventrolateral stripes. *H. orientalis* is smaller, with a green dorsum marked by fragmented orange stripes and distinct squared or round black spots and a mostly black ventral side with paired light green markings. *H. troctina* exhibits a green coloration with zigzag black lateral stripes and a broad posterior sucker (Trontelj and Utevsky 2012). *H. sulukii* is smaller, with elliptic black spots on the dorsolateral areas and two zigzag black stripes (Saglam et al. 2016). *H. nipponia* from East Asia has five thin dotted yellow longitudinal lines on a greenish dorsum and a uniform grey‐green ventral surface (Cheng et al. 2023). Finally, *H. tianjinensis* shows a green‐black dorsum with five continuous yellow stripes and pale ventral striping alongside a specific reproductive morphology (Wang et al. 2022).

The latest phylogenetic studies have clarified species boundaries, highlighting previously unresolved species structuring within *Hirudo*. These point to a Eurasian origin of the *Hirudo* ancestor, with an early split between the eastern lineage and Western Palearctic clades (Hechtel and Sawyer 2002; Trontelj and Utevsky 2012; Ceylan et al. 2021). Moreover, integrative taxonomy across the Iberian Peninsula, Maghreb and Sardinia further refined morphology, ecology and barcoding for western European species (Trontelj and Utevsky 2005; Arias et al. 2021).

For *H. verbana* specifically, phylogenetic studies revealed high genetic diversity and a population structure consistent with multiple Balkan and east European refugia based on mitochondrial lineages (Trontelj and Utevsky 2012; Popa et al. 2024). Currently, the most commercially widespread *H. verbana* has been confused for a long time with *H. medicinalis* (Siddall et al. 2007) and samples obtained in farms from the United Kingdom, France, Germany and the United States often have traceable roots in southeastern European and Turkish gene pools (Kutschera and Elliott 2014). From a conservation perspective, *H. verbana* remains relatively widespread across Europe compared to *H. medicinalis* , though its natural populations are increasingly fragmented and under pressure (Popa et al. 2024). Historical overexploitation for medicinal bloodletting, particularly during the 18th and 19th centuries, most likely caused severe declines in natural populations of medicinal leeches due to unsustainable harvesting, together with the previously mentioned confusion between species (Elliott and Kutschera 2011; Utevsky et al. 2014).

*Hirudo medicinalis* has been listed as ‘Near Threatened’ (Utevsky et al. 2014), due to habitat degradation, wetland drainage and historical overharvesting, Such assessment is currently only available for *H. verbana* in Romania, where it is classified as ‘vulnerable’. *H*. *verbana* retains relatively high levels of genetic diversity, particularly in southeastern Europe and the Balkans, which serve as biodiversity hotspots and potential refugia (Trontelj and Utevsky 2012; Popa et al. 2024). Maintaining this genetic variability is critical not only for long‐term species resilience but also for sustaining its role in biomedical and ecological research.

Starting from the *H*. *verbana* example, in this work we therefore set out to investigate the population structure of leech species across their natural ranges to better understand the origins of the genus. Moreover, the population fragmentation that has previously been recorded may, in only a few generations, lead to reduced gene flow and the gradual erosion of genetic diversity through drift and inbreeding. Such a process would be particularly concerning for medicinal leeches, which have already experienced sharp population declines due to overharvesting (Elliott 2008; Utevsky et al. 2014), habitat loss and commercial exploitation. In managing the remaining populations, it is therefore crucial to preserve genetic variability and to consider both natural dispersal routes and potential translocations that mimic historical patterns of gene flow. Molecular tools are becoming increasingly indispensable in this respect, providing the basis for effective conservation strategies that complement ecological and physiological studies.

The aims of this work are, therefore, multiple:

1. To analyse both cythochrome oxidase I (*COX1*) sequence and complete mitochondrial genomes to resolve the phylogenetic structure of *Hirudo* species and clarify their evolutionary relationships.
2. To apply molecular markers and Bayesian coalescent approaches to assess variability and divergence times among populations, providing insights into their historical demography.
3. To verify species boundaries within the *Hirudo* complex and confirm the status of recently described taxa while identifying previously unresolved lineages.
4. To produce the most comprehensive phylogeny to date of medicinal leeches and establish a time‐calibrated evolutionary framework that can guide taxonomy, conservation and biomedical applications

## 2 Materials and Methods

### 2.1 Collection and Extraction of Analysed Samples

We received a total of 36 leeches: eight from France (Ricarimpex, Eysines, France), eight from Lithuania (UAB LODEKSA, Liucinavo, Lithuania), 10 from Poland (EuroBion Paweł Drabczyński, Opole, Poland); these were all from aquacultures with a possible Eastern European origin and 10 from Turkey (Orderleeches.eu, the Netherlands) (Table S1). These were classified morphologically in our lab by two experts as *H. verbana*. Moreover, 10 specimens of leeches were collected in the wild from three different sites in Italy. Two sites were in the Campania region and one in the Lombardy region. Leeches were manually collected by actively searching in freshwater habitats at night, and specimens were chosen based on external morphological features suggestive of their tentative in‐field assignment to the genus *Hirudo* (see Table S1 for details and coordinates); however, only a small fragment of tissue was sent to us in ethanol, making proper morphological analysis impossible. Genomic DNA was extracted individually from each leech using the DNeasy Blood and Tissue Kit (Qiagen, Hilden, Germany), following the manufacturer's protocol specific for animal tissues or via a modified phenol‐chloroform protocol (Zuffi et al. 2022). A portion of the mitochondrial cytochrome c oxidase subunit I (*COX1*) gene was amplified either using the universal primers LCO1490 and HCO2198 (Folmer et al. 1994) or *H. verbana*‐specific primers designed for this work HiVer8099F (TCAATTTCGCATGTAGGCTGA) and HiVer9432R (TCTACATCTAGCCCAACCGT). PCR conditions followed the thermal cycling protocol described by Otim et al. (2021). The resulting amplicons were purified and sequenced in both directions (Eurofins Genomics GmbH, Konstanz, Germany). A final 596 bp *COX1* alignment was used for phylogenetic analyses.

### 2.2 In Silico Sample Acquisition

In addition to our samples, we searched the Sequence Read Archive (SRA) for genomic, transcriptomic or meta‐transcriptomic sequences belonging to any *Hirudo* species. We obtained 95 accessions in total: 15 *H. medicinalis* , 39 *H. nipponia*, 4 *H. orientalis* , 4 *H. tianjinensis*, 32 *H. verbana* and 1 *Hirudo* spp. Finally, all deposited *COX1* of *Hirudo* sequences from GenBank were added for a total of 230 accessions. *Limnatis nilotica* was used as an outgroup together with three additional genera, namely *Dina*, *Haemopis* and *Hirudinaria* (Tables S2 and S3).

### 2.3 Mitochondrial Read Extraction and Mapping

Complete mitogenome reads were extracted from downloaded reads using a modified in‐house pipeline (Naro et al. 2025). This pairs reads, removes duplicates and aligns to an available reference sequence, in this case, KU672397 (Nikitina et al. 2016) after making sure all genes were correct producing both the complete mitochondrial DNA and *CYB* sequences in fasta format. Accessions with only a single read file were firstly loaded into Geneious Prime v.2024.0.5 (https://www.geneious.com, Dotmatics, Boston MA) and filtered by removing duplicate sequences using Dedupe from BBTools, then aligned and mapped to an available leech mitogenome (KU672397) sequence using the Geneious mapper. Variant calling was performed using the Geneious algorithm when at least 70% of reads shared a mutation, otherwise they were considered heteroplasmies. An exception was made for node defining mutations when they were expected. The mitochondrial DNA map of *H. orientalis* was obtained using the Proksee server and Prokan Tools (Grant et al. 2023) was used for both GC content and GC skew. GC content is plotted as the deviation from the average GC content of the entire sequence, using a 500 bp sliding window. Positive and negative GC skews are relative to average GC content.

### 2.4 Phylogenetic Analyses and Age Estimates of mtDNA Haplogroups

The *COX1* and complete mtDNA maximum parsimony (MP) trees were built using Mega12.0.11, running 10,000 bootstrap replicates with the Subtree–Pruning–Regrafting method. The haplotype network was constructed using Fitchi v1.1.4 (Matschiner 2016). Age estimates of haplogroups were determined via Bayesian estimations using Beast v.2.7.8 (Bouckaert et al. 2019) under the HKY (*κ* = 2, estimated frequencies) nucleotide substitution model (8γ‐distributed rates, 1 shape and estimated proportion invariant sites) (as in Broggini et al. 2024) after confirming the correct substitution scheme with JModeltest v2.1.10 (Darriba et al. 2012). We used an Optimised Relaxed Clock (ORC), Log Normal set at a mean of 0.015 substitutions/site/Myr (Papadopoulou et al. 2010), confirmed by estimates based on three different methods which compare mitochondrial gene partition mutation rates (Broggini et al. 2024). A log normal distribution for the root height was set at 50 Myr (Bomfleur et al. 2015). 50 million MCMC chains were run with samples drawn every 1000 simulations after a burn‐in of 10%. Coalescence ages were obtained in two different runs, species level ages were obtained in the aforementioned Beast2 run, while *H. verbana* specific sub‐haplogroup dates were obtained in a second run using a strict clock normal distribution with mean 0.018 (*σ* 1.5515E^−4^), obtained in the previous run. Bayesian skyline plots were constructed using median tree heights and 2000 bins using tracer 1.7.3 (Rambaut et al. 2018). Haplogroup expansion maps were built using Surfer v 19.1.189 (Golden Software Inc., Golden, CO, USA, www.goldensoftware.com/) using the point kriging method and overlayed on a world map.

### 2.5 Ethics Approval Statement

This invertebrate species is not subject to ethical or regulatory restrictions.

## 3 Results

### 3.1 *H. verbana* Genetic Diversity

**FIGURE 1:**
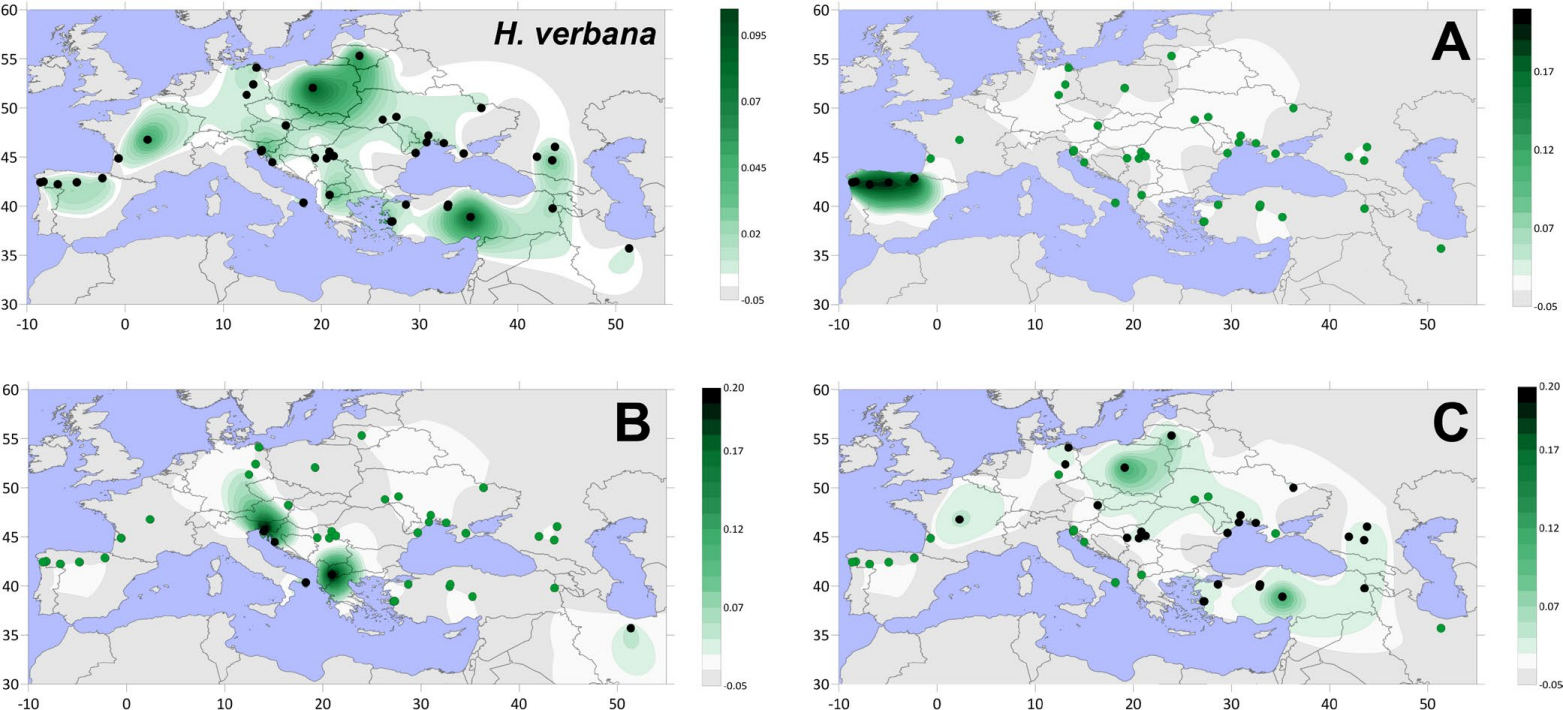
Spatial frequency distribution maps of all *Hirudo verbana* haplogroups and subhaplogroups. Dots indicate the geographical locations of the sampled individuals; black dots are specific of given haplogroup. Colour scale indicates frequency of haplogroup in the given map. The first panel is the distribution and *Hirudo verbana* as a species and its density. The remaining panels represent the main haplogroup density distributions. Panel letters correspond to the haplogroup name. Maps were generated with Surfer program (v 29.3.307, Golden Software Inc., Golden, CO, USA, www.goldensoftware.com/).

We obtained 43 *COX1* leech sequences from five countries (Table S1). These were combined with 247 GenBank entries and 60 sequences extracted from WGS reads, totalling 350 sequences. A total of 33 sequences (from Turkey, France, Lithuania and Poland) clustered with *H. verbana* into three main haplogroups (A–C) (Figure 1; Figure S1). These haplogroups correspond to the previously described Iberian, Western and Eastern clades (Trontelj and Utevsky 2012; Kutschera and Elliott 2014; Arias et al. 2021) (Figure S2). The Iberian clade (haplogroup A) comprises two geographically distinct sub‐haplogroups: A1, from north‐western Spain, and A2, from northern Spain (Figure 1A). The Western clade (haplogroup B) is represented mainly by sub‐haplogroup B1, distributed in Slovenia and Croatia, with additional samples from the southern Balkans (Figure 1B). The Eastern clade (haplogroup C) contains six sub‐haplogroups (C0–C5) spanning the species' Eastern‐most distribution (Figure 1C). Sub‐haplogroup C0, found in western Turkey, is basal to the others. C1 is the most widespread, occurring across eastern Europe and the Black Sea region, with a notable presence in French‐derived samples. C2 is restricted to the Aegean area of western Turkey. C3 occurs in imported American samples and one Turkish specimen. C4 is concentrated around the western Black Sea and western Ukraine, while C5 is mainly found in central Turkey and the eastern Black Sea, with additional individuals from a French breeding facility. Italian samples, on the other hand, were found to cluster together with *Haemopis sanguisuga* into two unpublished potential new species/subspecies and also a sister group to *Dina nesemanni* (described in detail in Supporting Information; while of general interest, these results are outside of the main scope of our work).

**FIGURE 2:**
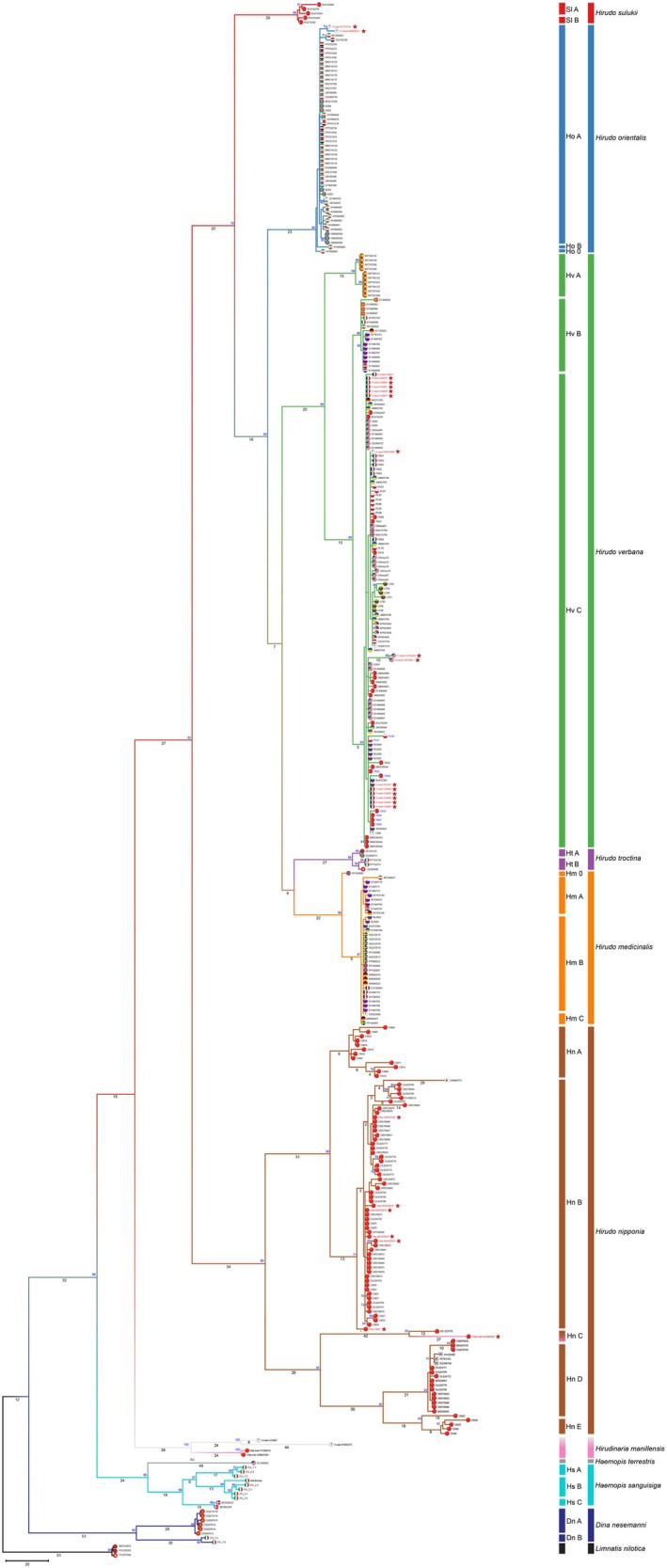
Maximum parsimony tree of *Hirudo* species, based on a *COX1* gene fragment. Numbers on branches indicate the number of nucleotide substitutions, and numbers at nodes indicate bootstrap values (all values below 50% are not shown). Colours represent the different species, and side bars indicate the different main haplogroups for each species. Flags indicate the country of origin of samples.

Within the 137 *H. verbana* sequences we found 111 segregating sites with 121 total mutations. These resulted in 50 different haplotypes with a haplotype diversity of 0.948 (±0.001) and a nucleotide diversity of 0.017 (±0.002). Within haplogroups, variability was found to be highest in A (2% ± 1.2%) and lowest in B (0.3% ± 0.1%), and between haplogroups we found that B and C are sister clades (*π* = 2.2% ± 0.2%) while haplogroup A has a substantial difference between both (~6.2% ± 1.2%) (Table S4). Analysis of the few available complete mitogenomes in GenBank, with the addition of 16 novel ones obtained in this work, is based on 22 samples which all correspond to *COX1* haplogroup C (inset of Figure S3). Here, two potential new sub‐haplogroups can be resolved from samples classified in COX1 phylogeny as simply ‘C’. Finally, we found that all Italian samples from this study belong to two other leech species; two specimens were found to be a sister clade to *Dina nasemanni* (Grosser et al. 2023, Figure S4) and the other eight were *Haemopis sanguisuga* (Figure 2).

### 3.2 Variability Within the *Hirudo* Genus

The *COX1* phylogeny of the complete genus *Hirudo* is in general agreement with the evolutionary trajectories suggested by Saglam et al. (2016), but provides a more resolved evolutionary framework. Within this topology, *H. verbana* forms a well‐supported clade with the *H. medicinalis–H. troctina* lineage. Together, these three taxa share a more recent common ancestor with *H. orientalis* . This larger assemblage is in turn closely related to the most recently described *H. sulukii*, while *H. nipponia* emerges as the earliest divergent lineage, occupying the basal position within the genus (Figure 2). Intraspecies variability (Table S5) was found to be highest in *H. nipponia* (*π* = 8.8% ± 0.8%) and lowest in *H. orientalis* (*π* = 0.15% ± 0.05%). Interspecies variability reflects the MP phylogeny, with an average of 73.6 nucleotide substitutions between species. Within *H. nipponia*, five main haplogroups (A–E) were identified, encompassing an overall variability of 8.8% (Table S6). Haplogroups A and B are closely related (~5% divergent) and form a well‐supported sister clade, whereas haplogroups C–E constitute a second lineage, with haplogroup C basal to haplogroups D and E (~7% divergent). The divergence between the A–B lineage and the C–E lineage is considerably higher (16%–20%).

In silico extraction of mitochondrial reads was employed to obtain entire mitogenomes for all other *Hirudo* species as well. We obtained 14 *H. medicinalis* (12 of which had *H. verbana* mitogenomes), 25 *H. nipponia*, one *H. tianjinensis* and four never before published *H. orientalis* (Figure S5). Median number of reads per sample was ~523,000; these were enough to cover roughly 98% of the mitogenome at 3000× coverage. Complete mitochondrial genomes provide a more resolved phylogenetic framework for these haplogroups (Figure S3). In this topology, haplogroup E is placed basal to the European and Middle Eastern lineages, while the placement of *H. sulukii* remains uncertain due to incomplete data. Haplogroup A diverges next, showing ~10% genetic distance from haplogroup E (Table S8). Haplogroup B is subdivided into two distinct clades, B and B1, with ~14% divergence between them. Haplogroup C, which also includes the reference sequence, occupies a more basal position relative to the aforementioned lineages, with an average divergence of ~24%. At the deepest level, haplogroup D represents the most basal lineage, separated by ~25% from the remaining clades. Samples identified as *H. tianjinensis* (Wang et al. 2022) cluster within haplogroup B and B1 in the *COX1* phylogeny, and specifically within B1 in the complete mitogenome tree.

### 3.3 Leech Phylogeny and Haplogroup Age Estimation

**FIGURE 3:**
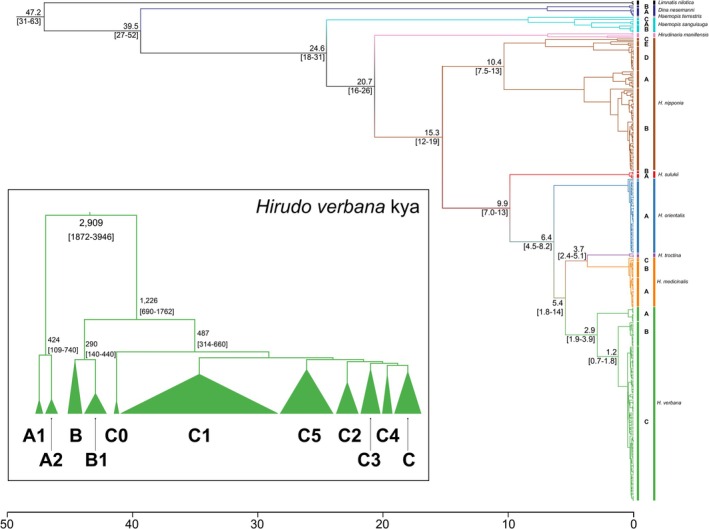
Temporal Bayesian reconstruction of Leech phylogeny. This consensus tree was obtained by running 50 million Markov Chain Monte Carlo (MCMC) chains (50,000 trees) and then removing 10% as burn‐in. It is based on the *COX1* fragment and encompasses 348 sequences from all available *Hirudo* species and closest outgroups. The tree was rooted using *Limnatis nilotica*. Colours are species‐specific; numbers at nodes indicate divergence date (in millions of years ago, Mya). The timeline at the bottom refers to the Bayesian coalescence times of Table S5. Inset, schematic Bayesian phylogeny; major sub‐haplogroups of *H. verbana* are represented as triangles which are proportional to the number of sequences present.

Using Bayesian molecular dating techniques implemented in beast2, we reconstructed the temporal framework of *Hirudo* diversification (Figure 3). The genus itself was estimated to originate in the mid‐Miocene, around 15.3 ± 3.6 Mya (Table S7), with an early split separating the East Asian *H. nipponia* from all other Eurasian species. Within *H. nipponia*, the major haplogroups are comparatively recent, with divergence times ranging from 0.36 to 1.85 Mya. The next branching event involved *H. sulukii*, which separated from the remaining taxa approximately 9.9 ± 2.9 Mya, while its extant lineages coalesce to around 350 ± 250 kya. The western Eurasian species form a distinct monophyletic clade dating to 6.38 ± 1.84 Mya. This group gave rise to two principal lineages: one leading to *H. orientalis* , which began differentiating in the Middle East around 443 ± 171 kya, and another encompassing the Western European species which diversified earlier, around 5.46 ± 1.59 Mya, the latter lineage subsequently splitting into *H. verbana* (2.91 ± 1.04 Mya) and the sister species *H. medicinalis* and *H. troctina* (3.60 ± 1.34 Mya). The most recent radiations within these lineages occurred during the late Pleistocene, with *H. verbana* emerging 276 ± 97 kya and *H. medicinalis* and *H. troctina* species radiating, respectively, 276 ± 97 kya and 409 ± 318 kya.

**FIGURE 4:**
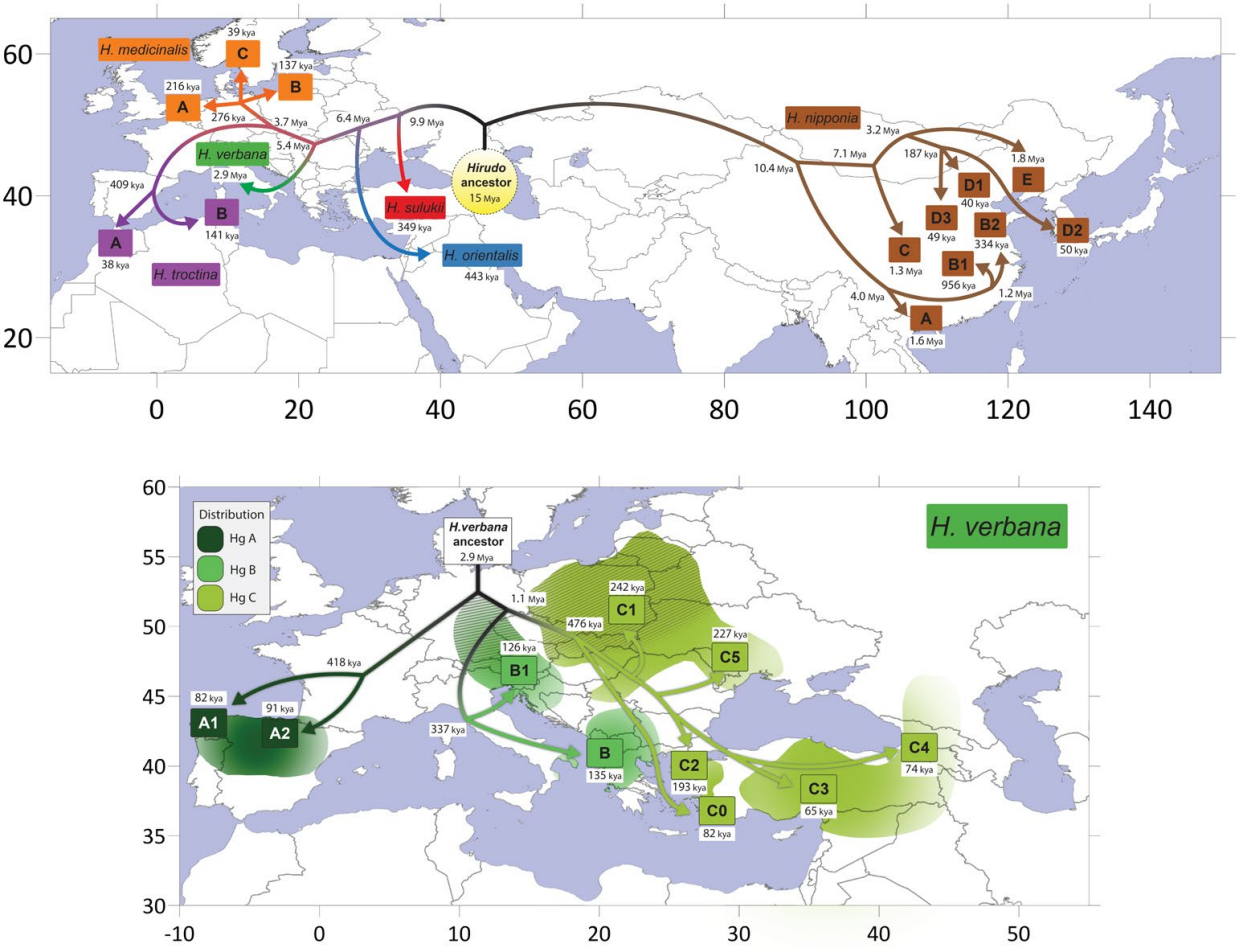
Postulated model for the geographical and temporal spread of leeches based on *COX1* segment. Map showing time divergence and species splits and diffusions of medicinal leeches. The yellow shaded circle in the top map indicates the possible time of origin of the medicinal leech ancestral mitogenome. Colours are species‐specific and boxed letters indicate major species haplogroup. The bottom map is specific to *Hirudo verbana* and its haplogroups. Distribution is relative to haplogroup distribution and lines in distribution indicate reintroduction/aquaculture areas. *Note:* The ancestor is placed in northern Europe just for simplicity of the diagram and *H. medicinalis* haplogroup A is placed further north for the same reason (samples are mainly from Slovenia, Croatia and Germany).

Our phylogeographic reconstruction points towards an eastern origin of the *H. verbana* ancestral mitogenome, followed by temporally structured splits and dispersal events that shaped the present‐day haplogroup distribution (Figures 4 and S6). Haplogroup A represents the earliest split, with current mitogenomes restricted to Spain and dating to 418 ± 160 kya. Its two sub‐lineages, A1 and A2, are comparatively recent, diverging ~82.5–90.6 kya. The separation between the eastern and western clades occurred 1.05 ± 0.23 Mya, followed by the differentiation of the Adriatic lineages (B and B1) between 126 and 337 kya. Eastern populations show greater diversification and occupy a broader distribution range. The most basal lineage (C0) is confined to Ankara (Turkey), while the Eastern European clade (C1) dates to 193 ± 46 kya and includes a geographically restricted Lithuanian subclade (C1d1, 101 ± 32 kya) of possible Turkish origin as suggested by Trontelj and Utevsky (2005). Additional clades are distributed across the Aegean (C2, 139 ± 48 kya) and around the Black Sea, where three major lineages occur: C3 (65 ± 33 kya), comprising American imports and a single Turkish sample, C4 (74 ± 37 kya, eastern Black Sea) and C5 (204 ± 68 kya, western Black Sea).

### 3.4 *H. verbana* Demography

**FIGURE 5:**
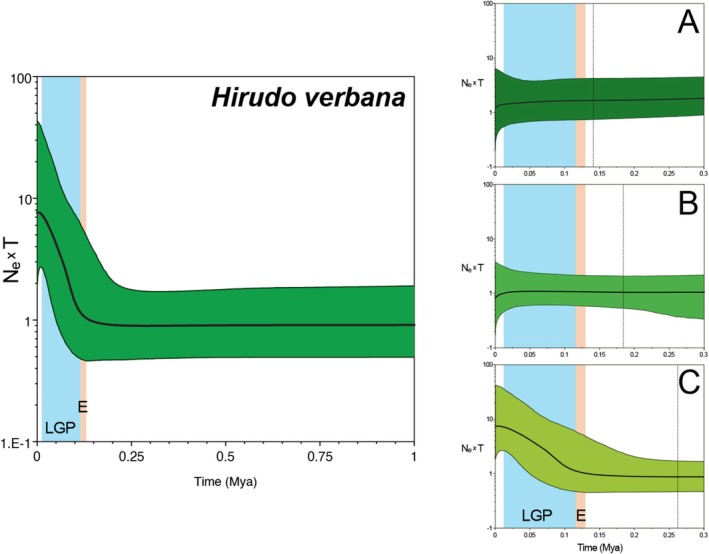
Bayesian Skyline Plot of *Hirudo verbana* COX1 sequences and its three main haplogroups. The darker line within the shaded areas indicates the median estimate of the effective population size × generation time and the coloured shading shows the 95% highest posterior density limits. The time axis in haplogroup BSPs is limited and normalised at 300 kya, beyond which the curve remains flat. Background colours represent: LGP, Last Glacial Period; E, Eamian (last interglacial). The vertical dotted line represents the boundary marker for the given MCMC sample subset.

The Bayesian Skyline Plot (Figure 5) shows the product of effective population size and generation time (*N*_e_ × *T*) over time for *H. verbana* and its haplogroups. There is only one major demographic event which occurred during the Last Glacial Period (LGP), here the population size increased 10‐fold from ~115 kya until today. Data extraction from haplogroups shows the same pattern.

## 4 Discussion

In this work, investigations on the population structure of several leech species across their natural ranges allowed us to shed light on the plausible origins of the *Hirudo* genus. Of note, the observed population fragmentation entails the risk of triggering a reduced gene flow and a progressive erosion of genetic diversity within this genus, thus emphasizing the need to preserve its genetic variability.

### 4.1 *H. verbana* Population Structure

Our phylogenetic analysis of partial *H. verbana COX1* sequences, including 59 new samples, reveals a distinct population structure that mirrors that previously reported (Trontelj and Utevsky 2012; Kutschera and Elliott 2014; Arias et al. 2021), confirming the robustness of these three major lineages (Iberian, Western and Eastern clades, from here on A, B and C, respectively). Whole mtDNA analysis unfortunately did not shed any additional light on haplogroup composition as all available whole mitogenomes (16 new ones) lie within haplogroup C only. Interestingly, 12 *H. medicinalis* (from France and Russia) sequences extracted from literature transcriptome reads cluster in haplogroup C; this has also been seen in a previous work (Siddall et al. 2007) where commercially available *H. medicinalis* sold as ‘medicinal leeches’ were in fact *H. verbana* (both new and previously described erroneous *H. medicinalis* are highlighted in red in Figure 2). The French samples were obtained from a *H. medicinalis* colony so the issue was most likely already addressed (Siddall et al. 2007) while the two Russian leeches (Babenko et al. 2020) were sampled in the Saratovskaya oblast, an area usually associated with *H. medicinalis* distribution; therefore erroneously classified.

The Iberian haplogroup A is largely restricted to northern Spain and both the phylogeny and nucleotide diversity point towards this group being distinct enough from the BC cluster to be considered a distinct subspecies (*Hirudo verbana bilineata*), thus confirming what was previously suggested (Arias et al. 2021). This clade separated ~2.9 Mya from the BC clade (Figure 4), a telling sign for a speciation event given the large separation time. Of note, ancestral dates could be even older if the entire mitogenome was considered. Extant Iberian lineages originated around 418 kya during the Middle Pleistocene glaciations and the distribution pattern is likely that of isolation from the main population in Iberian glacial refugia, corresponding to the previously described Picos de Europa and Ebro basin refugia (Gómez and Lunt 2007). This would suggest a previous, much larger and distributed breeding range spanning from the Iberian Peninsula all the way to the Caspian Sea. On the other hand, haplogroups B and C diverged 1.1 Mya during the Calabrian stage of the Pleistocene. Haplogroup B major haplogroups separated ~337 kya and, like haplogroup A, most likely had its original distribution range reduced to what it is today during the Middle Pleistocene glaciations (MIS9–10 transition period). Prior to this date, the Adriatic Sea's level was ~100–120 m (Grant et al. 2014; Rohling et al. 2014) lower due to the glaciations exposing a vast plain between Italy and the Balkans, which could explain the current distribution around the Adriatic Sea and Apennines. Subsequently (during MIS9), for roughly 30k years there was a warm interglacial period and the sea level of the Adriatic Sea rose to current day levels, separating the land masses. Haplogroups B1 and B diversified ~126 and ~135 kya, respectively, during the Penultimate Glacial Period; here the sea levels decreased again but not as much to form a complete land bridge. In fact, temperature variation in the Balkans and eastern Italy were not as extreme as the rest of Europe, once again leaving these areas as a glacial refuge (Van Andel and Tzedakis 1996). Haplogroup C exhibits the highest genetic diversity, the broadest geographic spread, and the greatest internal structuring of all the lineages indicating this as the ancestral pool of origin of the species. The ancestral lineage, C0, today restricted to western Turkey, diverged from the rest around 476 kya during the Elster Glaciation (MIS 12) and later split ~82 kya. This suggests that *H. verbana* dispersed and diversified over time giving rise to the major haplogroups now present in its non‐native area. Glacial–interglacial cycles likely facilitated this movement, as postglacial corridors such as the Dacian Basin opened migration routes toward Anatolia (Popa et al. 2024). Furthermore, repeated regressions of the Marmara and Black Seas during glacial low stands (Hoyle et al. 2021) may have provided additional pathways for colonisation into Turkey.

### 4.2 Climate Driven Speciation in *Hirudo*

On a much larger scale, the evolutionary history of the genus *Hirudo* appears to be closely linked to Neogene and Quaternary climactic shifts. Our Bayesian framework suggests that the common ancestor to *Hirudo* lived around 15 Mya around today's Transcaucasus, between the Black and the larger at the time, Caspian Seas. This is in concordance with an earlier hypothesis, which linked the area to greater genetic diversity and species richness (Kutschera and Shain 2019). The first major divergence in the phylogeny corresponds to the eastward dispersion and later expansion of *H. nipponia* ~10 Mya. Given our temporal estimate, this migration occurred while the Tibetan Plateau was still relatively lower in elevation compared to today (Wang et al. 2008), with Miocene paleo‐rivers providing freshwater corridors for a continuous distribution across central Asia. Moreover, the movement of hosts along this ancient ‘silk‐road’ of sorts could have fuelled the eastern dispersion. Enforced isolation occurred once the Tibetan Plateau and the Himalayas fully raised and the Gobi Desert became dry, establishing a long‐term barrier (Lu et al. 2019). Concurrently, lineages in the West diversified in the Mediterranean–Ponto‐Caspian region during the late Miocene–Pliocene. Climatic oscillations of the Pleistocene further restructured distributions, repeatedly fragmenting populations. In the Caucasus, *H. sulukii* differentiated from the European–Middle Eastern clade within the glacial freshwater refugium ~349 kya (MIS10 glaciation) present at the time (Saglam et al. 2016; Alçiçek et al. 2023). The split between *H. orientalis* and the European group occurred ~6.4 Mya, with secondary differentiation during the Middle Pleistocene (Elster Glaciation, MIS12), at the cusp of the relatively warm MIS11 interglacial (Milker et al. 2013). Within Europe, *H. verbana* diverged from the ancestor of *H. medicinalis* and *H. troctina* ~5.4 Mya, followed by subsequent separations among these three lineages. Postglacial expansions left clear phylogeographic signatures: *H. medicinalis* shows a south–north chrono‐gradient consistent with recolonisation from Adriatic refugia (Lambeck et al. 2010), while *H. troctina* colonised North Africa and Sardinia (the species is also present in Spain from where it most likely originated though no sequences are present in GenBank) during Late Pleistocene climatic fluctuations (Arias et al. 2021; Marrone et al. 2024).

### 4.3 *H. Nipponia,* More Than Just a Species

Among all *Hirudo* species, *H. nipponia* stands out for its rather high genetic diversity. Within species variability (Table S4) indicates deep divergences among the five main haplogroups which exceed the *COX1* threshold usually applied for species‐level recognition (Trontelj and Utevsky 2005; Siddall et al. 2007). Rather than a single panmictic species, we suggest that H. nipponia likely represents a complex of several cryptic taxa. Whole mitogenome data further reinforce this claim and reveal that the main haplogroups present within this clade actually occupy phylogenetically basal positions. Lineages such as C and D are so divergent that they appear closer to Hirudinaria than to the other sequences. This raises the possibility that previously published sequences available in the GenBank could be misidentifications (Xu et al. 2016). Moreover, the recent description of H. tianjinensis (Wang et al. 2022) within haplogroup B further highlights the taxonomic complexity. Together, these findings suggest that the diversity currently subsumed under the name H. nipponia may in fact represent multiple independently evolving species, shaped by long‐term climatic barriers and regional host movements. Additional genomic sampling across East Asia will be essential to clarify the true species boundaries and to reconcile taxonomic assignments with evolutionary history.

## 5 Conclusions

By incorporating the largest dataset of *Hirudo* samples assembled to date and applying molecular approaches, our work provides the most resolved evolutionary framework for the genus *Hirudo* to date, combining broad geographic sampling with mitogenomic data and Bayesian divergence dating. We confirm three major *H. verbana* clades shaped by Pleistocene glaciations and refugia, reveal potential cryptic diversity within *H. nipponia*, and document misidentified Italian leeches belonging to other genera. By establishing coalescent times and refining phylogenetic relationships, our work highlights the dynamic interplay of climate oscillations, geography and human exploitation in shaping the diversity of medicinal leeches.

These findings emphasise the critical importance of taxonomic clarity and genetic monitoring as foundational tools to support effective conservation strategies. Given the ecological roles of *Hirudo* species in freshwater ecosystems and their continued medical relevance, preserving their genetic diversity is essential for maintaining resilient populations amid environmental changes and anthropogenic pressures. Furthermore, clear taxonomic delineation aids in preventing overexploitation and mismanagement that may threaten population viability. Thus, integrating molecular insights with conservation policy not only sustains the biodiversity of these ecologically and medically important annelids but also ensures their sustainable use in biomedical and ecological applications.

Our results therefore underscore the need for taxonomic clarity and genetic monitoring to support both conservation efforts and the sustainable use of these ecologically and medically important species.

## Funding

This research was funded by grant FAR 2025 of the University of Insubria to G.B. and N.B.

## Conflicts of Interest

The authors declare no conflicts of interest.

## Supporting information


**Figure S1:** Haplotype network of *COX1* sequences showing the three main haplogroups (A–C). Sizes of circles are proportional to the number of sequences, with the smallest circle representing one individual. Dots on branches represent intermediate haplotypes. This figure was obtained with Fitchi v1.1.4.
**Figure S2:** Maximum parsimony tree of *Hirudo verbana* leeches based on *COX1*. Mutations on branches indicate number of nucleotide substitutions while blue numbers at nodes indicate bootstrap values. Colour shading indicates one of three main haplogroups.
**Figure S3:** Maximum parsimony of complete *Hirudo* mitogenomes. Mutations on branches indicate number of nucleotide substitutions while blue numbers at nodes indicate bootstrap values, flags indicate country of origin of samples. The green triangle represents all *H. verbana* samples. These can be seen more clearly in the inset.
**Figure S4:** Maximum parsimony tree of *Dina* species. Mutations on branches indicate number of nucleotide substitutions while blue numbers at nodes indicate bootstrap values.
**Figure S5:** Map of *H. orientalis* mitogenome. This map refers to the consensus of the four sequences (AZ01‐04). Genes are represented as blocks of different colours. Genes are indicated in orange. tRNAs are labelled according to single‐letter abbreviations. Direction of gene transcription is indicated by arrows (they are all encoded in + strand). The GC content is plotted using a black sliding window, as the deviation from the average GC content of the entire sequence. Positive and negative GC skews are relative to the average GC content of the entire sequence.
**Figure S6:** Spatial frequency distribution maps of *H. verbana* sub‐haplogroups of C. Dots indicate the geographical locations of the sampled individuals; black dots are specific of given haplogroup. Colour scale indicates frequency of haplogroup in the given map. Maps were generated with Surfer program (v 29.3.307, Golden Software Inc., Golden, CO, USA, www.goldensoftware.com/).
**Table S2:** *COX1* nucleotide diversity (%) of *Haemopis* species and haplogroups. Numbers in bottom left half‐matrix are the average number of mutations between specified species or haplogroups.
**Table S3:** *COX1* nucleotide diversity (%) of *Dina* main haplogroups. Numbers in bottom left half‐matrix are the average number of mutations between specified species.
**Table S4:** *COX1* nucleotide diversity (%) of *H. verbana* main haplogroups.
**Table S5:** *COX1* leech nucleotide diversity (%). Numbers in bottom left half‐matrix are the average number of mutations between specified species.
**Table S6:** *COX1* Nucleotide diversity (%) of *Hirudo nipponia* main haplogroups. Numbers in bottom left half‐matrix are the average number of mutations between specified haplogroups.
**Table S8:** Entire mitogenome nucleotide diversity (%) of *Hirudo nipponia* main haplogroups. Numbers in bottom left half‐matrix are the average number of mutations between specified haplogroups.

**Table S1:** Samples analysed for this study.

**Table S7:** Coalescence age estimates of *Hirudo* species and haplogroups using *COX1*. Bayesian age estimates for leech haplogroups, and clade separation ages of all available species in the genus.

## Data Availability

The data that support the findings of this study are openly available in GenBank at https://www.ncbi.nlm.nih.gov/nuccore with accession numbers: PX519676–PX519679; PX353594–PX353696.

## References

[eva70273-bib-0001] Alçiçek, H. , M. Gross , J. M. Bouchal , et al. 2023. “Paleobiodiversity and Paleoenvironments of the Eastern Paratethys Pleistocene Lacustrine‐Palustrine Sequence in the Baklan Basin (SW Anatolia, Turkey).” Palaeogeography, Palaeoclimatology, Palaeoecology 626: 111649. 10.1016/j.palaeo.2023.111649.

[eva70273-bib-0002] Arias, A. , V. Surugiu , R. Carballeira , O. P. Popa , L. O. Popa , and S. Utevsky . 2021. “Unravelling the Extent of Diversity Within the Iberian Medicinal Leeches (Hirudinea: *Hirudo*) Using Molecules and Morphology.” Biology 10, no. 4: 315. 10.3390/biology10040315. 33918739 PMC8070045

[eva70273-bib-0003] Babenko, V. V. , O. V. Podgorny , V. A. Manuvera , et al. 2020. “Draft Genome Sequences of *Hirudo medicinalis* and Salivary Transcriptome of Three Closely Related Medicinal Leeches.” BMC Genomics 21, no. 1: 331. 10.1186/s12864-020-6748-0. 32349672 PMC7191736

[eva70273-bib-0004] Baranzini, N. , L. Pulze , F. Acquati , and A. Grimaldi . 2020. “ *Hirudo verbana* as an Alternative Model to Dissect the Relationship Between Innate Immunity and Regeneration.” Invertebrate Survival Journal 17: 90–98. 10.25431/1824-307X/isj.v0i0.90-98.

[eva70273-bib-0005] Baranzini, N. , L. Pulze , C. Bon , et al. 2022. “ *Hirudo verbana* as a Freshwater Invertebrate Model to Assess the Effects of Polypropylene Micro and Nanoplastics Dispersion in Freshwater.” Fish & Shellfish Immunology 127: 492–507. 10.1016/j.fsi.2022.06.043. 35803505

[eva70273-bib-0006] Boidin‐Wichlacz, C. , D. Vergote , C. Slomianny , N. Jouy , M. Salzet , and A. Tasiemski . 2012. “Morphological and Functional Characterization of Leech Circulating Blood Cells: Role in Immunity and Neural Repair.” Cellular and Molecular Life Sciences 69, no. 10: 1717–1731. 10.1007/s00018-011-0897-x. 22159559 PMC11115165

[eva70273-bib-0007] Bomfleur, B. , T. Mörs , M. Ferraguti , M. A. Reguero , and S. McLoughlin . 2015. “Fossilized Spermatozoa Preserved in a 50‐Myr‐Old Annelid Cocoon From Antarctica.” Biology Letters 11, no. 7: 20150431. 10.1098/rsbl.2015.0431. 26179804 PMC4528455

[eva70273-bib-0008] Bouckaert, R. , T. G. Vaughan , J. Barido‐Sottani , et al. 2019. “BEAST 2.5: An Advanced Software Platform for Bayesian Evolutionary Analysis.” PLoS Computational Biology 15, no. 4: e1006650. 10.1371/journal.pcbi.1006650. 30958812 PMC6472827

[eva70273-bib-0009] Broggini, C. , M. Cavallini , I. Vanetti , J. Abell , G. Binelli , and G. Lombardo . 2024. “From Caves to the Savannah, the Mitogenome History of Modern Lions ( *Panthera leo* ) and Their Ancestors.” International Journal of Molecular Sciences 25, no. 10: 5193. 10.3390/ijms25105193. 38791233 PMC11121052

[eva70273-bib-0010] Ceylan, M. , R. Küçükkara , İ. Erbatur , E. Karataş , M. Tunç , and N. Sağlam . 2021. “Growth, Survival and Reproduction of the Turkish Medicinal Leech, *Hirudo Sulukii* .” Invertebrate Reproduction & Development 65, no. 1: 57–68. 10.1080/07924259.2021.1885506.

[eva70273-bib-0011] Cheng, B. , S. Kuang , G. Shao , et al. 2023. “Molecular Cloning and Functional Analysis of HnSaratin From *Hirudo nipponia* .” Gene 869: 147401. 10.1016/j.gene.2023.147401. 36996929

[eva70273-bib-0012] Darriba, D. , G. L. Taboada , R. Doallo , and D. Posada . 2012. “jModelTest 2: More Models, New Heuristics and Parallel Computing.” Nature Methods 9, no. 8: 772. 10.1038/nmeth.2109. PMC4594756 22847109

[eva70273-bib-0013] Elliott, J. M. 2008. “Population Size, Weight Distribution and Food in a Persistent Population of the Rare Medicinal Leech, *Hirudo medicinalis* .” Freshwater Biology 53, no. 8: 1502–1512. 10.1111/j.1365-2427.2008.01978.x.

[eva70273-bib-0014] Elliott, J. M. , and U. Kutschera . 2011. “Medicinal Leeches: Historical Use, Ecology, Genetics and Conservation.” Freshwater Reviews 4, no. 1: 21–41. 10.1608/FRJ-4.1.417.

[eva70273-bib-0055] Folmer, O. , M. Black , W. Hoeh , R. Lutz , and A. R. Vrijenhoek . 1994. “DNA Primers for Amplification of Mitochondrial Cytochrome c Oxidase Subunit I From Diverse Metazoan Invertebrates.” Molecular Marine Biology and Biotechnology 3, no. 5: 294–299. 7881515

[eva70273-bib-0016] Gómez, A. , and D. H. Lunt . 2007. “Refugia Within Refugia: Patterns of Phylogeographic Concordance in the Iberian Peninsula.” In Phylogeography of Southern European Refugia: Evolutionary Perspectives on the Origins and Conservation of European Biodiversity, edited by S. Weiss and N. Ferrand , 155–188. Springer Netherlands. 10.1007/1-4020-4904-8_5.

[eva70273-bib-0017] Grant, J. R. , E. Enns , E. Marinier , et al. 2023. “Proksee: In‐Depth Characterization and Visualization of Bacterial Genomes.” Nucleic Acids Research 51, no. W1: W484–W492. 10.1093/nar/gkad326. 37140037 PMC10320063

[eva70273-bib-0018] Grant, K. M. , E. J. Rohling , C. B. Ramsey , et al. 2014. “Sea‐Level Variability Over Five Glacial Cycles.” Nature Communications 5, no. 1: 5076. 10.1038/ncomms6076. 25254503

[eva70273-bib-0019] Grosser, C. , T. Rewicz , M. Jovanović , A. Zawal , and V. Pešić . 2023. “Integrative Taxonomy Reveals a New Species of the Leech Genus *Dina* R. Blanchard, 1892 (Annelida, Hirudinida: Erpobdellidae) From the Ancient Skadar Lake Basin in Montenegro.” European Zoological Journal 90, no. 1: 383–394. 10.1080/24750263.2023.2216710.

[eva70273-bib-0021] Hechtel, F. O. , and R. T. Sawyer . 2002. “Toward a Taxonomic Revision of the Medicinal Leech *Hirudo medicinalis* Linnaeus, 1758 (Hirudinea: Hirudinidae): Re‐Description of *Hirudo troctina* Johnson, 1816 From North Africa.” Journal of Natural History 36, no. 11: 1269–1289. 10.1080/00222930110048945.

[eva70273-bib-0022] Hildebrandt, J. P. , and S. Lemke . 2011. “Small Bite, Large Impact–Saliva and Salivary Molecules in the Medicinal Leech, *Hirudo medicinalis* .” Naturwissenschaften 98, no. 12: 995–1008. 10.1007/s00114-011-0859-z. 22069059

[eva70273-bib-0023] Hoyle, T. M. , D. Bista , R. Flecker , W. Krijgsman , and F. Sangiorgi . 2021. “Climate‐Driven Connectivity Changes of the Black Sea Since 430 Ka: Testing a Dual Palynological and Geochemical Approach.” Palaeogeography, Palaeoclimatology, Palaeoecology 561: 110069. 10.1016/j.palaeo.2020.110069.

[eva70273-bib-0024] Kutschera, U. , and J. Elliott . 2014. “The European Medicinal Leech *Hirudo medicinalis* L.: Morphology and Occurrence of an Endangered Species.” Zoosystematics and Evolution 90, no. 2: 271–280. 10.3897/zse.90.8715.

[eva70273-bib-0025] Kutschera, U. , and D. S. Shain . 2019. “Hirudinea Lamarck 1818: Evolutionary Origin and Taxonomy of the Six Medicinal Leeches (Genus *Hirudo*) Known Today.” Biomedical Research and Reviews 3, no. 1: 1–4. 10.15761/BRR.1000126.

[eva70273-bib-0026] Lambeck, K. , A. Purcell , J. Zhao , and N. O. Svensson . 2010. “The Scandinavian Ice Sheet: From MIS 4 to the End of the Last Glacial Maximum.” Boreas 39, no. 2: 410–435. 10.1111/j.1502-3885.2010.00140.x.

[eva70273-bib-0027] Lemke, S. , and A. Vilcinskas . 2020. “European Medicinal Leeches—New Roles in Modern Medicine.” Biomedicine 8, no. 5: 99. 10.3390/biomedicines8050099. PMC7277884 32349294

[eva70273-bib-0028] Lu, H. , X. Wang , X. Wang , et al. 2019. “Formation and Evolution of Gobi Desert in Central and Eastern Asia.” Earth‐Science Reviews 194: 251–263. 10.1016/j.earscirev.2019.04.014.

[eva70273-bib-0029] Marrone, F. , F. Stoch , L. Vecchioni , M. M. Botta , S. Utevsky , and F. P. Faraone . 2024. “On the Occurrence of the Dragon Leech *Hirudo troctina* Johnson, 1816 (Annelida, Hirudinea) in Sardinia (Italy).” European Zoological Journal 91, no. 2: 842–854. 10.1080/24750263.2024.2378831.

[eva70273-bib-0030] Matschiner, M. 2016. “Fitchi: Haplotype Genealogy Graphs Based on the Fitch Algorithm.” Bioinformatics 32, no. 8: 1250–1252. 10.1093/bioinformatics/btv717. 26656006

[eva70273-bib-0031] Milker, Y. , R. Rachmayani , M. F. G. Weinkauf , et al. 2013. “Global and Regional Sea Surface Temperature Trends During Marine Isotope Stage 11.” Climate of the Past 9, no. 5: 2231–2252. 10.5194/cp-9-2231-2013.

[eva70273-bib-0032] Montinari, M. R. , and S. Minelli . 2022. “From Ancient Leech to Direct Thrombin Inhibitors and Beyond: New From Old.” Biomedicine & Pharmacotherapy 149: 112878. 10.1016/j.biopha.2022.112878. 35364378

[eva70273-bib-0033] Moquin‐Tandon, A. 1846. Monographie de la famille des Hirudinées, 328–340. J.‐B. Baillière. 10.5962/bhl.title.6598.

[eva70273-bib-0034] Naro, G. , G. Lombardo , A. Alvaro , et al. 2025. “Discovery of a New Inland Population of *Leptoconops noei* in Italy With Sequencing of the First Complete Mitochondrial Genome for the Genus.” Medical and Veterinary Entomology 39, no. 4: 817–828. 10.1111/mve.12828. 40641369 PMC12586292

[eva70273-bib-0035] Nikitina, A. , V. Babenko , T. Akopian , et al. 2016. “Draft Mitochondrial Genomes of *Hirudo medicinalis* and *Hirudo verbana* (Annelida, Hirudinea).” Mitochondrial DNA Part B Resources 1, no. 1: 254–256. 10.1080/23802359.2016.1157774. 33473467 PMC7800963

[eva70273-bib-0036] Otim, M. H. , S. Adumo Aropet , M. Opio , D. Kanyesigye , H. Nakelet Opolot , and W. Tek Tay . 2021. “Parasitoid Distribution and Parasitism of the Fall Armyworm *Spodoptera frugiperda* (Lepidoptera: Noctuidae) in Different Maize Producing Regions of Uganda.” Insects 12, no. 2: 121. 10.3390/insects12020121. 33573080 PMC7912086

[eva70273-bib-0037] Papadopoulou, A. , I. Anastasiou , and A. P. Vogler . 2010. “Revisiting the Insect Mitochondrial Molecular Clock: The Mid‐Aegean Trench Calibration.” Molecular Biology and Evolution 27, no. 7: 1659–1672. 10.1093/molbev/msq051. 20167609

[eva70273-bib-0038] Popa, O. P. , A. Ștefan , E. Ș. Baltag , A. A. Stratan , L. O. Popa , and V. Surugiu . 2024. “High Genetic Diversity of *Hirudo verbana* Carena, 1820 (Annelida: Hirudinea: Hirudinidae) in Romania Confirms That the Balkans Are Refugia Within Refugium.” Diversity 16, no. 12: 726. 10.3390/d16120726.

[eva70273-bib-0039] Rambaut, A. , A. J. Drummond , D. Xie , G. Baele , and M. A. Suchard . 2018. “Posterior Summarization in Bayesian Phylogenetics Using Tracer 1.7.” Systematic Biology 67, no. 5: 901–904. 10.1093/sysbio/syy032. 29718447 PMC6101584

[eva70273-bib-0040] Rohling, E. J. , G. L. Foster , K. M. Grant , et al. 2014. “Sea‐Level and Deep‐Sea‐Temperature Variability Over the Past 5.3 Million Years.” Nature 508, no. 7497: 477–482. 10.1038/nature13230. 24739960

[eva70273-bib-0041] Saglam, N. , R. Saunders , S. A. Lang , and D. H. Shain . 2016. “A New Species of *Hirudo* (Annelida: Hirudinidae): Historical Biogeography of Eurasian Medicinal Leeches.” BMC Zoology 1, no. 1: 5. 10.1186/s40850-016-0002-x.

[eva70273-bib-0042] Siddall, M. E. , P. Trontelj , S. Y. Utevsky , M. Nkamany , and K. S. Macdonald III . 2007. “Diverse Molecular Data Demonstrate That Commercially Available Medicinal Leeches Are Not *Hirudo medicinalis* .” Proceedings of the Royal Society B: Biological Sciences 274, no. 1617: 1481–1487. 10.1098/rspb.2007.0248. PMC2176162 17426015

[eva70273-bib-0043] Trontelj, P. , and S. Y. Utevsky . 2005. “Celebrity With a Neglected Taxonomy: Molecular Systematics of the Medicinal Leech (Genus *Hirudo*).” Molecular Phylogenetics and Evolution 34, no. 3: 616–624. 10.1016/j.ympev.2004.10.012. 15683933

[eva70273-bib-0044] Trontelj, P. , and S. Y. Utevsky . 2012. “Phylogeny and Phylogeography of Medicinal Leeches (Genus *Hirudo*): Fast Dispersal and Shallow Genetic Structure.” Molecular Phylogenetics and Evolution 63, no. 2: 475–485. 10.1016/j.ympev.2012.01.022. 22342869

[eva70273-bib-0045] Utevsky, S. , M. Zagmajster , A. Atemasov , et al. 2010. “Distribution and Status of Medicinal Leeches (Genus *Hirudo*) in the Western Palaearctic: Anthropogenic, Ecological, or Historical Effects?” Aquatic Conservation: Marine and Freshwater Ecosystems 20, no. 2: 198–210. 10.1002/aqc.1071.

[eva70273-bib-0046] Utevsky, S. , M. Zagmajster , and P. Trontelj . 2014. *Hirudo medicinalis* . IUCN Red List of Threatened Species. https://www.iucnredlist.org/species/pdf/21415816.

[eva70273-bib-0047] Van Andel, T. H. , and P. C. Tzedakis . 1996. “Palaeolithic Landscapes of Europe and Environs, 150,000‐25,000 Years Ago: An Overview.” Quaternary Science Reviews 15, no. 5–6: 481–500. 10.1016/0277-3791(96)00028-5.

[eva70273-bib-0048] Wagenaar, D. A. 2015. “A Classic Model Animal in the 21st Century: Recent Lessons From the Leech Nervous System.” Journal of Experimental Biology 218, no. 21: 3353–3359. 10.1242/jeb.113860. 26538172

[eva70273-bib-0049] Wang, C. , X. Zhao , Z. Liu , et al. 2008. “Constraints on the Early Uplift History of the Tibetan Plateau.” Proceedings of the National Academy of Sciences 105, no. 13: 4987–4992. 10.1073/pnas.0703595105. PMC2278176 18362353

[eva70273-bib-0050] Wang, H. , F. M. Meng , S. J. Jin , J. W. Gao , X. R. Tong , and Z. C. Liu . 2022. “A New Species of Medicinal Leech in the Genus *Hirudo* Linnaeus, 1758 (Hirudiniformes, Hirudinidae) From Tianjin City, China.” ZooKeys 1095: 83. 10.3897/zookeys.1095.74071. 35836684 PMC9021146

[eva70273-bib-0051] Wang, W. , Y. Liu , W. Lou , et al. 2025. “A Comprehensive Quality Evaluation System for Medicinal Leeches by Integrating Macromolecular Protein Analysis and Small‐Molecule Marker Detection as Well as Quantitative Bioassays.” Pharmaceuticals 18, no. 6: 887. 10.3390/ph18060887. 40573282 PMC12195895

[eva70273-bib-0052] Weiskopf, S. R. , K. P. McCarthy , M. Tessler , et al. 2018. “Using Terrestrial Hematophagous Leeches to Enhance Tropical Biodiversity Monitoring Programmes in Bangladesh.” Journal of Applied Ecology 55, no. 4: 2071–2081. 10.1111/1365-2664.13111.

[eva70273-bib-0053] Xu, Y. , J. Nie , J. Hou , L. Xiao , and P. Lv . 2016. “Complete Mitochondrial Genome of *Hirudo nipponia* (Annelida, Hirudinea).” Mitochondrial DNA Part A DNA Mapping, Sequencing, and Analysis 27, no. 1: 257–258. 10.3109/19401736.2014.883614. 24521495

[eva70273-bib-0054] Zuffi, M. A. , A. J. Coladonato , G. Lombardo , et al. 2022. “The Italian Wall Lizard, *Podarcis siculus campestris*, Unexpected Presence on Gorgona Island (Tuscan Archipelago).” Acta Herpetologica 17: 135–145. 10.36253/a_h-12388.

