# Supplementary material for: Blood Relatives: Linking Evolutionary History and Conservation of Medicinal Leeches (Hirudo spp.)
Source: Evol Appl. 2026 May 28;19(6):e70273. doi: 10.1111/eva.70273 (PMC13238757; doi:10.1111/eva.70273)
Supplement: Supplementary file 1 — Figure S1: Haplotype network of COX1 sequences showing the three main haplogroups (A–C). Sizes of circles are proportional to the number of sequences, with the smallest circle representing one individual. Dots on branches represent intermediate haplotypes. This figure was obtained with Fitchi v1.1.4. Figure S2: Maximum parsimony tree of Hirudo verbana leeches based on COX1. Mutations on branches indicate number of nucleotide substitutions while blue numbers at nodes indicate bootstrap values. Colour shading indicates one of three main haplogroups. Figure S3: Maximum parsimony of complete Hirudo mitogenomes. Mutations on branches indicate number of nucleotide substitutions while blue numbers at nodes indicate bootstrap values, flags indicate country of origin of samples. The green triangle represents all H. verbana samples. These can be seen more clearly in the inset. Figure S4: Maximum parsimony tree of Dina species. Mutations on branches indicate number of nucleotide substitutions while blue numbers at nodes indicate bootstrap values. Figure S5: Map of H. orientalis mitogenome. This map refers to the consensus of the four sequences (AZ01‐04). Genes are represented as blocks of different colours. Genes are indicated in orange. tRNAs are labelled according to single‐letter abbreviations. Direction of gene transcription is indicated by arrows (they are all encoded in + strand). The GC content is plotted using a black sliding window, as the deviation from the average GC content of the entire sequence. Positive and negative GC skews are relative to the average GC content of the entire sequence. Figure S6: Spatial frequency distribution maps of H. verbana sub‐haplogroups of C. Dots indicate the geographical locations of the sampled individuals; black dots are specific of given haplogroup. Colour scale indicates frequency of haplogroup in the given map. Maps were generated with Surfer program (v 29.3.307, Golden Software Inc., Golden, CO, USA, www.goldensoftware.com/). Ta [file EVA-19-e70273-s001.docx]

**Blood Relatives: Linking Evolutionary History and Conservation of Medicinal Leeches (Hirudo spp.)**

Gianluca Lombardo, Alessandro Alvaro, Andrea De Benedictis, Marta Cavallini, Milo Manica, Edward C. Netherlands, Laura Pulze, Nicolò Baranzini, Annalisa Grimaldi, Francesco Acquati, Giorgio Binelli.

Supplementary material

## Italian leeches

The ten sampled Italian leeches clustered within the *Haemopis sanguisuga* phylum in two distinct haplogroups, named A and B (Supplementary Table S6). The remaining, haplogroup C, is Denmark-specific, indicating distinct population structure. Nucleotide diversity values for the *COX1* fragments show ~6.3% divergence between all haplogroups; this value far exceeds the ~2-3% intraspecies divergence rate used for barcoding species (Hebert *et al*., 2003), indicating haplogroups A and B are either cryptic or a never-before sequenced species present in Italy. In depth morphological analyses could further confirm this hypothesis. The remaining two Italian sequences (IT_1.2 and IT_1.9) showed 89.82% and 89.92% identity, respectively, with *Dina nesemanni* (Supplementary Figure S4), a species recently described from Montenegro (Grosser *et al.*, 2023). Nucleotide diversity values of all available *COX1* sequences further supports this conclusion (Supplementary Table S5), with the Italian samples having an inter species nucleotide diversity value of *π* = 10.5 ± 4.2% with *D. nesemanni* and increasing diversity with other *Dina* species. This value is comparable to the between-species diversity of *D. nesemanni* and *D. latestriata* (π = 12.2 ± 4.2%), therefore indicating that the Italian samples could indeed represent a new species. In Italy, *D. lineata* (Müller, 1774) and, according solely to FaunaItalia (https://www.faunaitalia.it/checklist/), *Dina apathy* Gedroyć, 1916 are the only species recorded for the genus (Minelli, 1979). Unfortunately, no COI sequences for *D. apathyi* are currently available in public databases, so we could not compare the sequences generated in our study with those of that species. Nevertheless, this species was described from Poland and is considered an Eastern-European species (Jueg and Zettler, 2015). Moreover, considering that the Mediterranean climate is similar in both our Campania sampling sites and the sites from which *D. nesemanni* was collected in Montenegro (the only known range of the species by now), it is unlikely that our specimens belong to *D. apathyi*. Instead, they probably represent a species new to science, related to *D. nesemanni*. Further morphological and ecological analyses are required to confirm this hypothesis and potentially describe a new species within the genus *Dina*.

Supplementary Figures


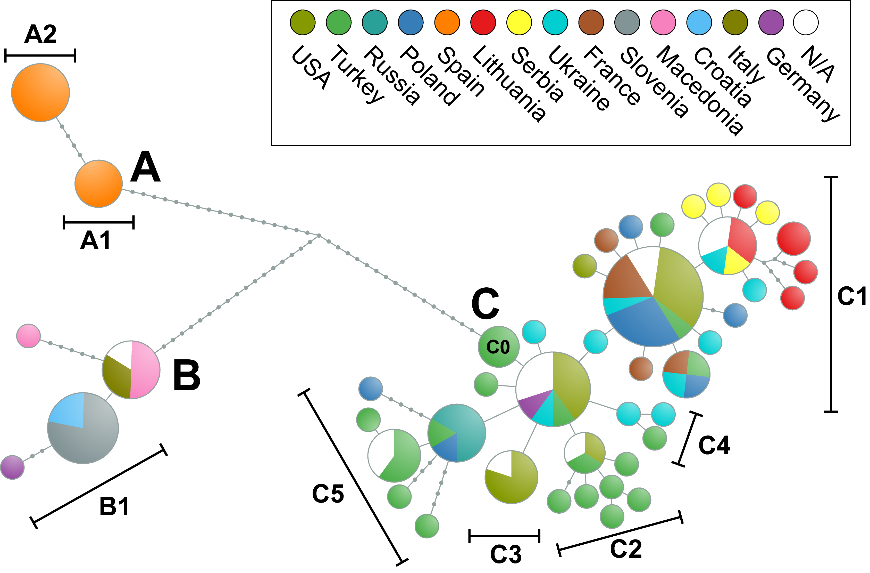


**Supplementary Figure 1: Haplotype network of *COX1* sequences showing the three main haplogroups (A-C).** Sizes of circles are proportional to the number of sequences, with the smallest circle representing one individual. Dots on branches represent intermediate haplotypes. This figure was obtained with Fitchi v1.1.4.

**
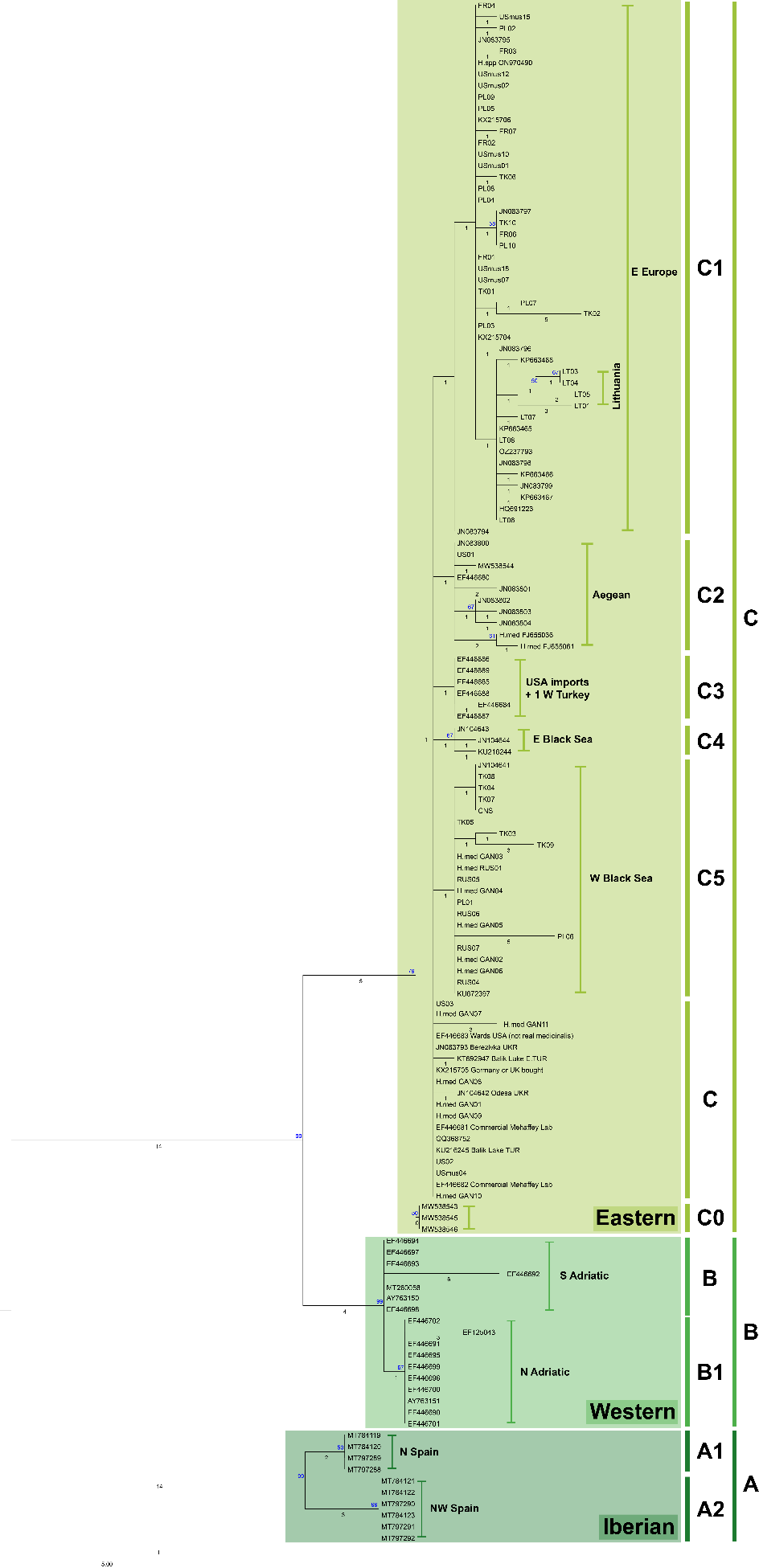
**

**Supplementary Figure S2: Maximum parsimony tree of *Hirudo verbana* leeches based on *COX1.*** Mutations on branches indicate number of nucleotide substitutions while blue numbers at nodes indicate bootstrap values. Colour shading indicates one of three main haplogroups.


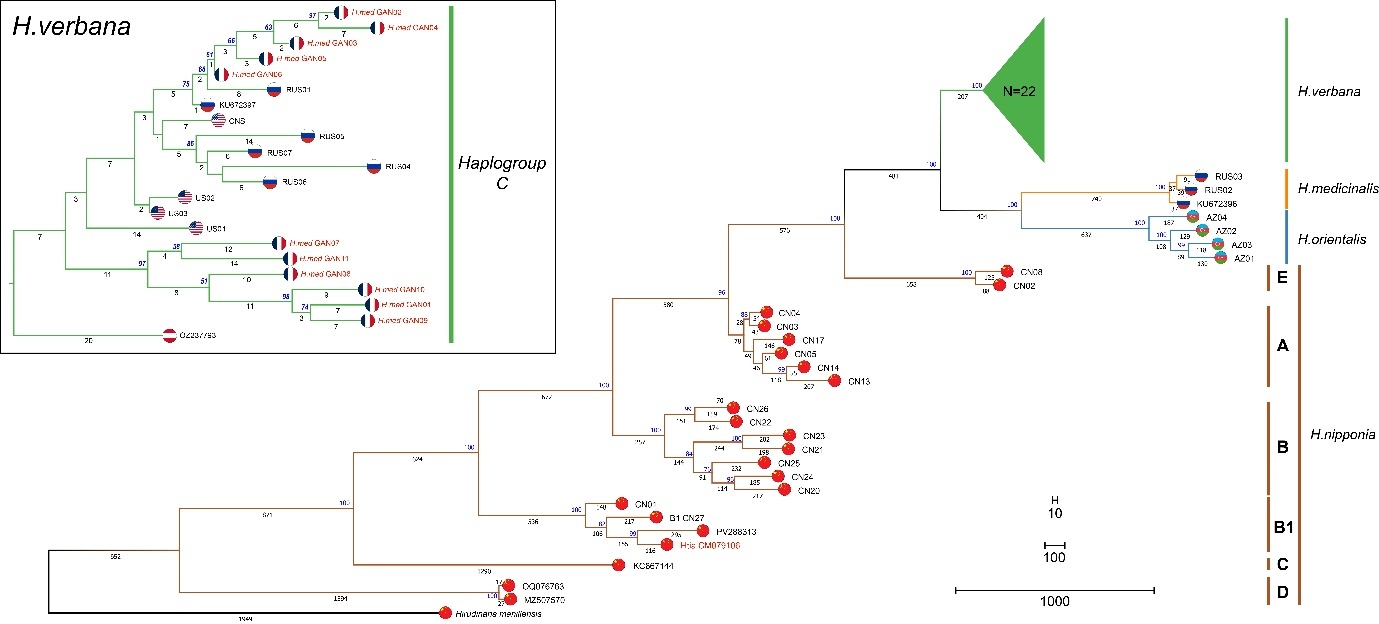


**Supplementary Figure S3: Maximum parsimony of complete *Hirudo* mitogenomes.** Mutations on branches indicate number of nucleotide substitutions while blue numbers at nodes indicate bootstrap values, flags indicate country of origin of samples. The green triangle represents all *H. verbana* samples. These can be seen more clearly in the inset.


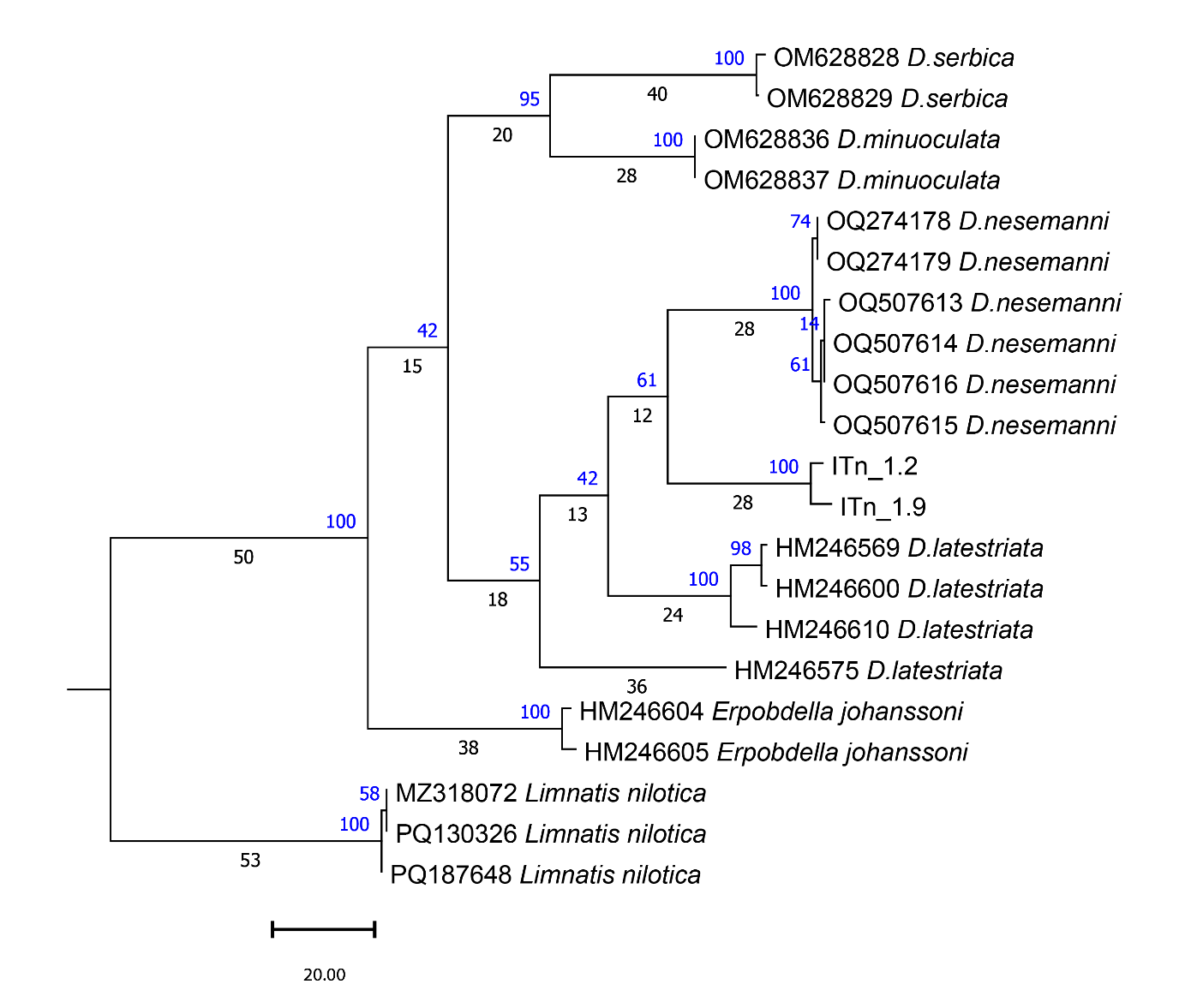


**Supplementary Figure S4: Maximum parsimony tree of *Dina* species.** Mutations on branches indicate number of nucleotide substitutions while blue numbers at nodes indicate bootstrap values.

**
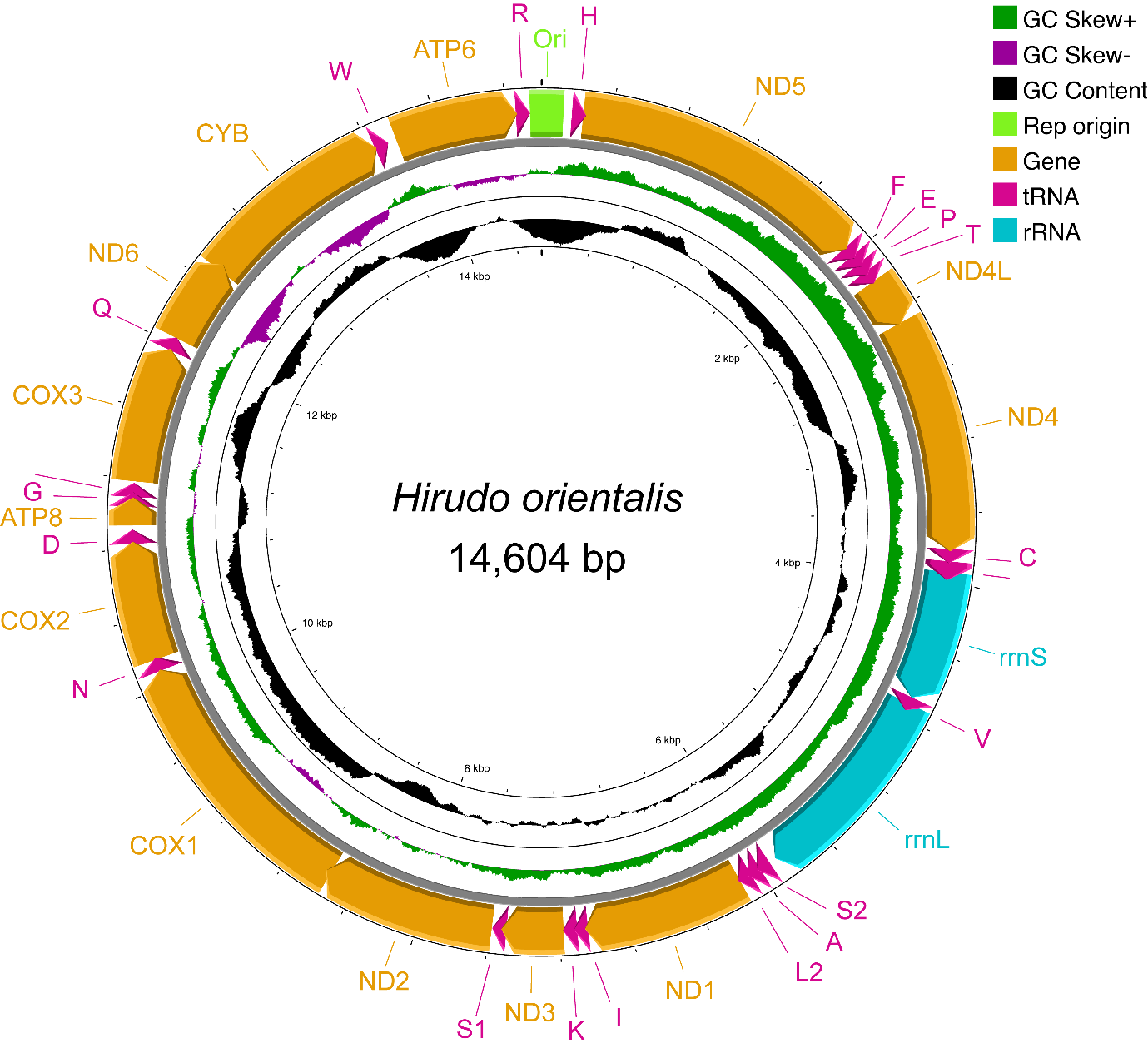
**

**Supplementary Figure S5: Map of *H. orientalis* mitogenome.** This map refers to the consensus of the 4 sequences (AZ01-04). Genes are represented as blocks of different colours. Genes are indicated in orange. tRNAs are labelled according to single-letter abbreviations. Direction of gene transcription is indicated by arrows (they are all encoded in + strand). The GC content is plotted using a black sliding window, as the deviation from the average GC content of the entire sequence. Positive and negative GC skews are relative to the average GC content of the entire sequence.

**
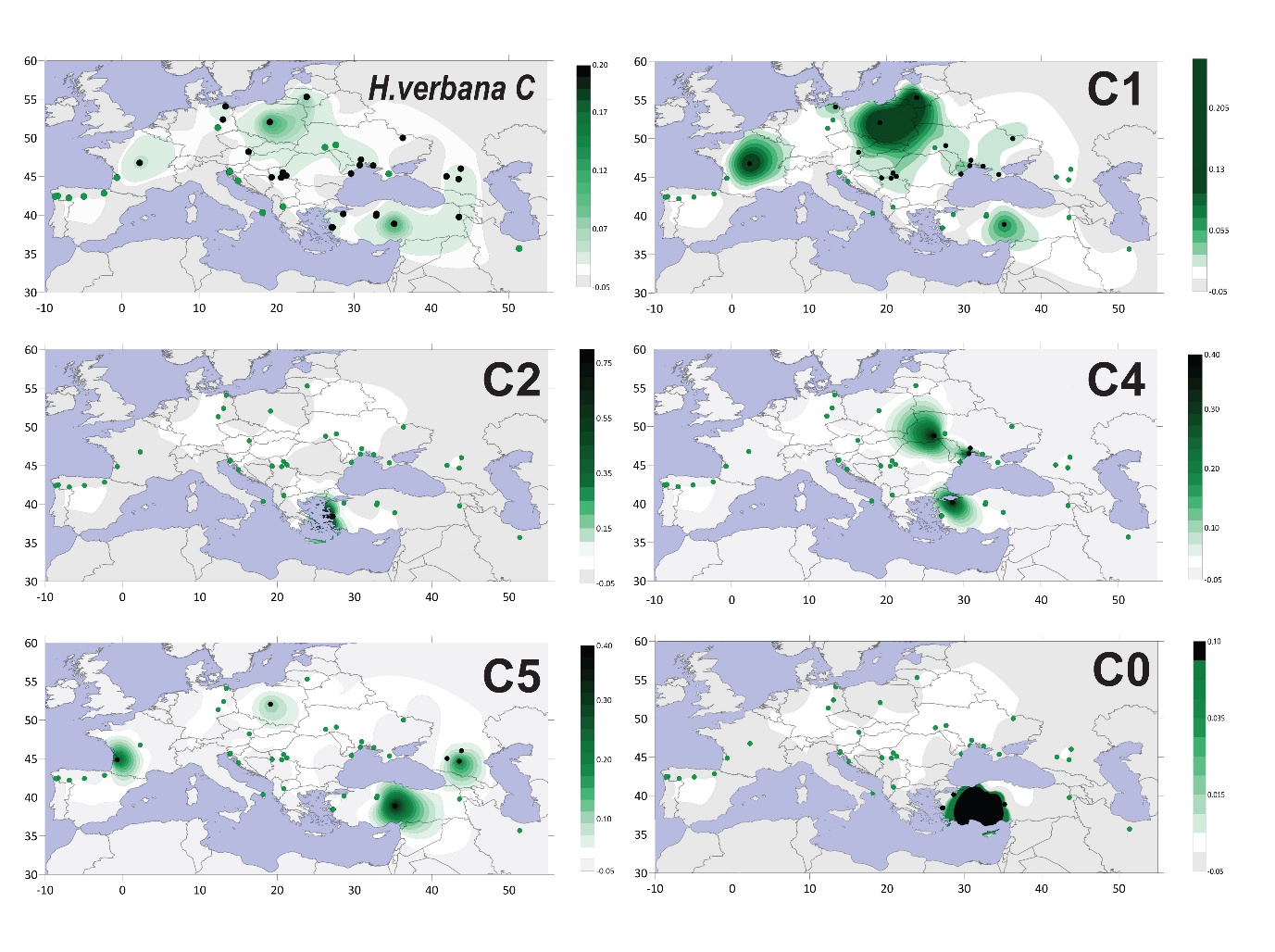
**

**Supplementary Figure S6: Spatial frequency distribution maps of *H. verbana* sub-haplogroups of C.** Dots indicate the geographical locations of the sampled individuals; black dots are specific of given haplogroup. Colour scale indicates frequency of haplogroup in the given map. Maps were generated with Surfer program (v 29.3.307, Golden Software, Inc., Golden, CO, USA, [www.goldensoftware.com/](http://www.goldensoftware.com/)).

Supplementary Tables

**Supplementary Table 1 – Samples analysed for this study**

EXCEL

**Supplementary Table 2 – COX1 Nucleotide diversity (%) of *Haemopis species and* haplogroups.** Numbers in bottom left half-matrix are the average number of mutations between specified species or haplogroups.

|  | *Hae ter* | *Hae san* | Hs-A | Hs-B | Hs-C |
| --- | --- | --- | --- | --- | --- |
| *Hae ter* | N/A | 12.6% | 13.0% | 12.6% | 12.0% |
| n=1 | N/A | 4.1% | 6.1% | 5.1% | 8.5% |
| Hae san | 69.1 | 4.8% | 4.6% | 3.8% | 5.1% |
| n=10 |  | 0.5% | 1.6% | 1.3% | 1.9% |
| Hs-A | 71.0 | 26.2 | 0.6% | 6.4% | 6.2% |
| n=3 |  |  | 0.2% | 2.0% | 3.3% |
| Hs-B | 69.2 | 21.9 | 36.7 | 1.6% | 6.4% |
| n=5 |  |  |  | 0.5% | 2.7% |
| Hs-C | 66.0 | 28.9 | 35.7 | 36.4 | N/A |
| n=2 |  |  |  |  | N/A |

**Supplementary Table 3 – COX1 Nucleotide diversity (%) of *Dina* main haplogroups.** Numbers in bottom left half-matrix are the average number of mutations between specified species.

| Dina | *D. nesemanni* | Italy This work | *D. serbica* | *D. minuoculata* | *D. latestriata* | All Dina | *Erpobdella johanssoni* |
| --- | --- | --- | --- | --- | --- | --- | --- |
| *D. nesemanni* | 0.3% | 10.5% | 19.3% | 16.5% | 12.2% | 8.7% | 15.7% |
| n=6 | 0.1% | 4.2% | 7.6% | 7.4% | 4.2% | 2.5% | 6.2% |
| Italy | 57.7 | 1.0% | 17.7% | 14.3% | 12.0% | 11.0% | 16.8% |
| n=2 |  | 0.5% | 8.9% | 8.8% | 5.3% | 3.0% | 8.4% |
| *D. serbica* | 101.2 | 93.0 | 0.3% | 12.4% | 17.0% | 15.3% | 17.6% |
| n=2 |  |  | 0.2% | 7.6% | 7.5% | 4.2% | 8.8% |
| *D.minuoculata* | 88.0 | 77.0 | 68.0 | 0.0% | 16.1% | 13.3% | 14.8% |
| n=2 |  |  |  | 0.0% | 8.5% | 4.0% | 9.1% |
| *D. latestriata* | 67.1 | 65.5 | 90.7 | 86.3 | 8.1% | 16.1% | 16.9% |
| n=4 |  |  |  |  | 4.1% | 8.5% | 7.4% |
| All Dina | 46.4 | 59.0 | 79.8 | 70.4 | 86.3 | 11.8% | 15.8% |
| n=16 |  |  |  |  |  | 1.2% | 3.7% |
| *E. johanssoni* | 84.3 | 89.0 | 93.5 | 80.0 | 90.0 | 83.7 | 0.7% |
| n=2 |  |  |  |  |  |  | 0.3% |

**Supplementary Table 4 – *COX1* Nucleotide diversity (%) of *H. verbana* main haplogroups.**

| *H. verbana* | **A** | **B** | **C** |
| --- | --- | --- | --- |
| **A** | 2.0% | 6.3% | 6.1% |
| n=11 | 1.2% | 1.5% | 0.8% |
| **B** | 36.0 | 0.3% | 2.2% |
| n=17 |  | 0.1% | 0.2% |
| **C** | 34.9 | 12.6 | 0.5% |
| n=109 |  |  | 0.0% |

Numbers in bottom left half-matrix are the average number of mutations between specified haplogroups.

**Supplementary Table 5 – COX1 Leech Nucleotide diversity (%).** Numbers in bottom left half-matrix are the average number of mutations between specified species.

| Species diversity | *H. verbana* | *H. medicinalis* | *H. orientalis* | *H. nipponia* | *H. sulukii* | *H. troctina* | *Hirudinaria manillensis* | *Haemopis terrestris* | *Haemopis sanguisuga* | *Dina* spp | *Limnatis* |
| --- | --- | --- | --- | --- | --- | --- | --- | --- | --- | --- | --- |
| *H. verbana* | 1.6% | 9.9% | 10.0% | 21.3% | 13.1% | 10.9% | 23.7% | 20.3% | 19.8% | 28.4% | 24.4% |
| n=136 | 0.2% | 0.6% | 0.7% | 0.7% | 1.5% | 1.6% | 4.2% | 4.9% | 1.6% | 2.6% | 3.6% |
| *H. medicinalis* | 55.1 | 0.3% | 8.9% | 20.3% | 10.9% | 9.4% | 22.6% | 19.8% | 18.0% | 26.9% | 23.6% |
| n=34 |  | 0.1% | 1.4% | 1.3% | 2.8% | 3.1% | 8.9% | 10.8% | 3.3% | 5.5% | 7.8% |
| *H. orientalis* | 55.6 | 50.0 | 0.1% | 19.1% | 12.1% | 10.0% | 22.8% | 20.2% | 19.3% | 27.8% | 24.6% |
| n=52 |  |  | 0.0% | 1.6% | 4.2% | 4.5% | 12.4% | 15.4% | 4.7% | 7.7% | 11.1% |
| *H. nipponia* | 107.2 | 102.6 | 97.6 | 8.8% | 19.5% | 19.1% | 18.1% | 19.2% | 20.4% | 29.6% | 25.4% |
| n=92 |  |  |  | 0.8% | 1.9% | 2.4% | 2.8% | 3.6% | 1.4% | 2.4% | 3.2% |
| *H. sulukii* | 71.7 | 60.2 | 66.6 | 99.5 | 0.9% | 13.0% | 23.1% | 21.5% | 18.3% | 28.1% | 25.5% |
| n=5 |  |  |  |  | 0.3% | 4.6% | 8.3% | 8.6% | 3.6% | 6.5% | 9.1% |
| *H. troctina* | 60.5 | 52.7 | 55.5 | 97.7 | 70.8 | 0.7% | 21.8% | 18.0% | 20.6% | 28.1% | 24.0% |
| n=3 |  |  |  |  |  | 0.3% | 11.4% | 10.9% | 5.6% | 8.9% | 11.9% |
| *Hd.manil* | 121.0 | 115.7 | 116.8 | 93.0 | 118.3 | 112.7 | 4.3% | 22.1% | 22.7% | 33.0% | 27.9% |
| n=2 |  |  |  |  |  |  | 2.2% | 11.1% | 6.5% | 11.1% | 14.5% |
| *Ha.terrestris* | 105.9 | 103.4 | 105.2 | 98.1 | 111.2 | 95.0 | 114.0 | N/A | 12.6% | 26.2% | 23.6% |
| n=1 |  |  |  |  |  |  |  | N/A | 4.1% | 10.6% | 14.4% |
| *Ha. Sangui* | 103.3 | 94.8 | 101.0 | 103.6 | 96.7 | 107.1 | 116.6 | 69.1 | 4.8% | 23.6% | 22.5% |
| n=10 |  |  |  |  |  |  |  |  | 0.5% | 4.1% | 6.1% |
| *Dina* spp | 139.7 | 133.2 | 136.9 | 140.4 | 138.4 | 138.4 | 157.4 | 130.5 | 119.5 | 4.7% | 24.2% |
| n=8 |  |  |  |  |  |  |  |  |  | 1.7% | 7.7% |
| *Limnatis* | 123.7 | 120.5 | 125.0 | 124.7 | 128.5 | 122.3 | 138.7 | 120.7 | 115.6 | 122.2 | 0.1% |
| n=3 |  |  |  |  |  |  |  |  |  |  | 0.1% |

**Supplementary Table 6 – COX1 Nucleotide diversity (%) of *Hirudo nipponia* main haplogroups.** Numbers in bottom left half-matrix are the average number of mutations between specified haplogroups.

|  | Nip-A | Nip-B | Nip-C | Nip-D | Nip-E | *H. nipponia* |
| --- | --- | --- | --- | --- | --- | --- |
| Nip-A | 2.0% | 5.4% | 19.6% | 20.4% | 17.3% | 8.5% |
| n=12 | 0.3% | 0.5% | 5.2% | 3.5% | 3.5% | 1.0% |
| Nip-B | 30.8 | 1.3% | 18.5% | 19.0% | 16.3% | 6.1% |
| n=58 |  | 0.2% | 3.8% | 1.7% | 2.2% | 0.8% |
| Nip-C | 102.1 | 97.0 | 4.3% | 16.9% | 17.8% | 18.0% |
| n=2 |  |  | 2.2% | 6.7% | 7.0% | 2.8% |
| Nip-D | 104.3 | 98.5 | 88.6 | 0.3% | 7.0% | 15.1% |
| n=17 |  |  |  | 0.1% | 2.0% | 1.4% |
| Nip-E | 91.3 | 86.7 | 94.1 | 38.9 | 3.4% | 14.4% |
| n=4 |  |  |  |  | 1.0% | 1.6% |
| *H. nipponia* | 45.6 | 32.1 | 93.2 | 78.0 | 75.6 | 8.8% |
| n=93 |  |  |  |  |  | 0.8% |

**Supplementary Table 7 – Coalescence age estimates of *Hirudo* species and haplogroups using *COX1*.** Bayesian age estimates for leech haplogroups, and clade separation ages of all available species in the genus.

EXCEL

**Supplementary Table 8 – Entire mitogenome nucleotide diversity (%) of *Hirudo nipponia* main haplogroups.** Numbers in bottom left half-matrix are the average number of mutations between specified haplogroups.

| mtDNA | Nip-A | Nip-B | Nip-C | Nip-D | Nip-E | *H.nipponia* |
| --- | --- | --- | --- | --- | --- | --- |
| Nip-A | 1.9% | 9.1% | 22.1% | 26.5% | 9.9% | 10.7% |
| n=6 | 0.3% | 1.0% | 7.4% | 8.8% | 3.3% | 2.2% |
| Nip-B | 1452.0 | 9.1% | 20.4% | 26.3% | 18.0% | 12.4% |
| n=11 |  | 1.0% | 5.3% | 6.7% | 4.8% | 1.8% |
| Nip-C | 2792.3 | 2604.6 | N/A | 26.8% | 26.2% | 20.2% |
| n=1 |  |  | N/A | 13.4% | 13.1% | 4.0% |
| Nip-D | 3258.6 | 3228.9 | 3291.5 | 0.3% | 20.5% | 23.6% |
| n=2 |  |  |  | 0.1% | 10.2% | 4.7% |
| Nip-E | 1347.8 | 2319.4 | 3224.8 | 2613.3 | 1.4% | 15.3% |
| n=2 |  |  |  |  | 0.7% | 3.3% |
| *H. nipponia* | 1410.8 | 1620.3 | 2540.7 | 2904.3 | 1972.7 | 14.5% |
| n=22 |  |  |  |  |  | 1.7% |
